# Molecular Docking Simulations Provide Insights in the Substrate Binding Sites and Possible Substrates of the ABCC6 Transporter

**DOI:** 10.1371/journal.pone.0102779

**Published:** 2014-07-25

**Authors:** Mohammad Jakir Hosen, Abdullah Zubaer, Simrika Thapa, Bijendra Khadka, Anne De Paepe, Olivier M. Vanakker

**Affiliations:** 1 Center for Medical Genetics, Ghent University Hospital, Ghent, Belgium; 2 Department of Genetic Engineering and Biotechnology, Shahjalal University of Science and Technology, Sylhet, Bangladesh; 3 Swapnojaatra Bioresearch Laboratory, DataSoft Systems, Dhaka, Bangladesh; University of Technology Sydney, Australia

## Abstract

The human ATP-binding cassette family C member 6 (ABCC6) gene encodes an ABC transporter protein (ABCC6), primarily expressed in liver and kidney. Mutations in the ABCC6 gene cause pseudoxanthoma elasticum (PXE), an autosomal recessive connective tissue disease characterized by ectopic mineralization of the elastic fibers. The pathophysiology underlying PXE is incompletely understood, which can at least partly be explained by the undetermined nature of the ABCC6 substrates as well as the unknown substrate recognition and binding sites. Several compounds, including anionic glutathione conjugates (N-ethylmaleimide; NEM-GS) and leukotriene C4 (LTC4) were shown to be modestly transported in vitro; conversely, vitamin K3 (VK3) was demonstrated not to be transported by ABCC6. To predict the possible substrate binding pockets of the ABCC6 transporter, we generated a 3D homology model of ABCC6 in both open and closed conformation, qualified for molecular docking and virtual screening approaches. By docking 10 reported in vitro substrates in our ABCC6 3D homology models, we were able to predict the substrate binding residues of ABCC6. Further, virtual screening of 4651 metabolites from the Human Serum Metabolome Database against our open conformation model disclosed possible substrates for ABCC6, which are mostly lipid and biliary secretion compounds, some of which are found to be involved in mineralization. Docking of these possible substrates in the closed conformation model also showed high affinity. Virtual screening expands this possibility to explore more compounds that can interact with ABCC6, and may aid in understanding the mechanisms leading to PXE.

## Introduction

ABCC6 (also known as Multidrug Resistance Protein-6, MRP6) is a transporter protein, belonging to the adenosine triphosphate (ATP)-binding cassette (ABC) family, primarily expressed in liver and kidney [1]. Perturbance of the ABCC6 transporter due to mutations in the encoding ABCC6 gene causes pseudoxanthoma elasticum (PXE), an autosomal recessive connective tissue disorder, affecting the skin, eyes and cardiovascular system, due to progressive mineralization and fragmentation of elastic fibers in the extracellular matrix [2]. Current evidence suggests PXE to be a metabolic disease, in which the defective ABCC6 protein fails to transport one or more metabolites from liver and kidney cells into the circulation [3], [4], [5], [6]. However, until now only in vitro substrates of the ABCC6 transporter have been identified, mainly glutathione conjugates, demonstrating ABCC6 to be an organic anion transporter [6], [7], [8]. Next to the speculation on the main physiological substrates, no structural data on possible substrate recognition and binding sites of ABCC6 are presently available.

As currently no high resolution X-ray crystallographic structure for ABCC6 is available, 3D homology modeling may be an alternative to gain insights into potential substrate binding sites and the mechanisms of substrate interaction with the protein [9]. A 3D configuration of a prokaryotic ABCC6 protein was successfully modeled using the X-ray structure of the Staphylococcus aureus Sav1866 export pump [10]. Fulop et al (2009) [11] used this model as a template to build an outward-facing conformation (that corresponds to the ATP-bound or closed conformation state) 3D homology model for human ABCC6 and have used this model and the distribution of PXE-causing mutations to demonstrate the relevance of the transmission interfaces as well as the ABC-ABC domain contacts for the function of the transporter. Varadi et al (2011) [12] were also able to build an ATP-free/wide open inward facing confirmation of this 3D model (http://www.enzim.hu/~varadi/ABCC6/).

In this study, we have first generated an ABCC6 homology model in open conformation, and applied molecular docking of known in vitro substrates of ABCC6, to evaluate if we could predict potential sites responsible for protein-substrate interaction. Based on the prediction of binding pockets in the ABCC6 transporter, we carried out a virtual screening in the open conformation ABCC6 model using the Human Serum Metabolome Database (HSMD) against these substrate binding sites of ABCC6, to generate a list of potential substrate molecules. In the second phase, we have generated a closed conformation homology model of ABCC6 to evaluate the reproducibility of the open conformation docking results. The compounds in this list with the highest binding energy are discussed on their potential role in PXE pathophysiology.

## Materials and Methods

### Homology modeling of ABCC6

The primary sequence of the human ABCC6 protein was retrieved from the SwissProt Protein database (UniProt Accession: O95255) which has a sequence length of 1503 amino acids. In order to find suitable template(s) for the ABCC6 protein, PSI-BLAST against all existing molecules in the Protein Data Bank (PDB) was performed [13], which revealed highest homology of ABCC6 with P-glycoprotein (P-gp, mouse ABCB1 transporter). Compared to P-gp, ABCC1 has a higher homology with ABCC6, but no PDB file of ABCC1 is listed in the PDB database. P-gp was therefore used as a template to generate an open conformation ABCC6 3D homology model. To generate the open conformation structure, initially four models which include amino acid residues 302 to 1503 of ABCC6 were generated using three Metaservers including I-TASSER [14] Genesilico [15] and Pcons [16], and the MODELLER v9.9 program [17]. Models obtained from I-TASSER, Genesilico, Pcons and MODELLER were then used again as templates to build a final model of ABCC6 using MODELLER 9.9. Secondary structure elements were revealed by Genesilico Metaserver. The loop regions of the final model were rebuilt using Modloop [18], and the energy minimization of the resulting final model was performed using the YASARA energy minimization server [19].

To meet the observation of a higher homology between ABCC6 and ABCC1 compared to P-gp, we generated a second homology model with ABCC1 as a template. Because of its absence in the PDB database, the template was based on the only available ABCC1 model reported by DeGorter et al. (2008), which is only in a closed conformation [20]. Because a 3D homology model always follows the spatial arrangement of the template, we could thus only generate a closed conformation ABCC6 model based on the ABCC1 template. The MODELLER v9.9 program and the template counterparts from 302–856 and 943–1503 were used to generate this closed conformation 3D homology model. In addition, a full length 3D structure of ABCC6 was generated using MODELLER v9.9 containing all 1503 amino acid residues with P-gp as a template.

### Model validation

The stereo-chemical quality of the final models were evaluated using three different servers (ERRAT [21], Rampage [22] and ProQ [23]), which are used to determine the statistical significance of a protein 3D model considering spatial position of amino-acids and overall stability of the structure. Usually more than 90% accuracy is expected for the validity of a model. Furthermore, template-target superposition based on structural alignment has been used in SuperPose [24] to understand the coverage between template and target-protein, and the reliability of the model.

### Topology and intrinsically disordered region prediction

The membrane topology of the open conformation ABCC6 protein was predicted using software from the PONGO server [25]. It uses four selected and high scoring predictors - MEMSAT, TMHMM2, PRODIV and ENSEMBLE 1.0, and the predicted result can be displayed in a graphical view. As TMHMM [26] has been considered the best transmembrane prediction method, we have used the TMHMM server (http://www.cbs.dtu.dk/services/TMHMM/) individually to assess the probability of the transmembrane domain position in the ABCC6 sequence (302–1503). Furthermore, the ABCC6 amino acid sequence (302–1503) was divided into four segments and submitted to the MetaDisorder server [27], a meta-server for the prediction of the intrinsically disordered region. Exclusion of these disordered regions was considered during generation of our ABCC6 models.

### Electrostatic charge distribution

Electrostatic potential surface (EPS) of both the open and closed conformation ABCC6 modeled protein including its substrate binding sites was calculated using PBEQ solver (http://www.charmm-gui.org/?doc=input/pbeqsolver) and resulted outputs (3D structure with surface potential scale of −10 Kcal/mol to +10 Kcal/mol) were displayed in PyMOL (http://www.pymol.org).

### Ligand molecules selection

Initially, 8 previously reported in vitro substrates (Table S1) were designed and validated to use as reference ligands to perform molecular docking. Ligand coordinates (2D structure; Figure S1) were obtained from the DrugBank [28], PubChem Compound database (http://pubchem.ncbi.nlm.nih.gov/) and ZINC database [29]. Second, vitamin K1 (VK1) and vitamin K2 (VK2) were evaluated as ligands, as well as vitamin K3 (VK3). The latter was used as a negative control, as it has been previously shown not to be transported by ABCC6 [30]. Third, after delineation of predicted binding site(s), all 4651 metabolites listed in the Human Serum Metabolome Database (HSMD; http://www.serummetabolome.ca/scripts/hmdbDownload.cgi) were used to dock our ABCC6 open conformation 3D homology model. The substrate molecules with no available coordinates were drawn and converted into 3D structure, and generation of mol-files was carried out using the ACD/ChemSketch software [31]. Energy minimization of these compounds was then performed using the United Force field (UFF) program [32].

### Molecular docking approach for the identification of substrate binding sites

To identify the substrate binding sites and possible binding pattern of 8 reported substrates and three vitamin K isoforms with the ABCC6 transporter protein, molecular docking was performed using Autodock 4.2 [33] and Autodock Vina [34]. In this study, a rigid docking protocol was considered in order to reduce the computational cost and time in which the receptor was kept rigid and ligands were allowed to rotate. For blind docking, in order to remove the bias, the whole protein transmembrane (TM) region was covered with a grid. The size of the grid box was set to 80 Å x 80 Å x 80 Å (x, y and z) with 0.925 Å spacing between the grid points. To search the best conformation space of the ligand, the Lamarckian Genetic Algorithm was employed. During the blind docking process, 100 different conformers were generated for each ligand with a population size of 150 individuals. A maximum number of evaluation was set to 2,500,000 and maximum number of generation to 27,000, while other parameters were kept as default.

A refined docking was then carried out in a specific area to optimize the results, for which the grid box size was also set to 80 Å×80 Å×80 Å (x, y and z) whereas the space between grid points was reduced to 0.55277 Å. For the docking process, the number of generation was reduced to 20 conformers for each ligand with a population size of 250 individuals. Maximum number of evaluation was set to 25,000,000 while maximum number of generation was set to 27,000. Other parameters were used as default. In both the initial blind docking and refined docking approach, selection of the final pose of ligand bound with the protein was done by giving priority to the lowest binding-energy conformation with largest binding-cluster of the total conformers.

A detailed analysis of residues involved in the interaction between ligands and protein was conducted using the Discovery Studio 3.1 Visualizer software (Accelyrs Software Inc., Discovery Studio Modeling Environment, and Release 3.1.San Diego: Accelrys Software Inc., 2012).

Manual inspection was employed to investigate the occurrence of mutations in substrate binding sites (SBS). To identify the number of naturally found mutations residing in SBS, the amino-acids involved in SBS were compared to the list of natural variants in ABCC6 at UniProt (http://www.uniprot.org/uniprot/O95255#section_features) and the unique mutations annotated in the Leiden Open Variation Database (LOVD) of the ABCC6 gene (http://www.ncbi.nlm.nih.gov/lovd/home.php?select_db=ABCC6).

### Virtual screening and docking

First, structure based virtual screening against HSMD was carried out with Autodock Vina (for each compound 10 bound conformations were generated). During virtual screening the docking was applied only to the substrate binding sites in the transmembrane region. For this, the grid box was set to 70 Å×70 Å×60 Å with default value of 1.0 Å spacing by Autodock Vina. The grid box was large enough to cover the whole predicted substrate binding region. Furthermore, Autodock was used to verify the docking results by re-docking the resulted compounds, and to analyze the interaction between top scoring compounds and the ABCC6 protein models.

## Results and Discussion

### Homology modeling of human ABCC6

To generate a homology model of the ABCC6 protein, we initially performed a PSI-BLAST search towards the PDB database against the ABCC6 sequence extracted from UniProt (AC no: O95255). Generally, identity of ≥30% between the target and template sequence is widely accepted for comparative modeling [35]. However, for membrane transporter proteins identity of ≥20–40% is expected to establish a protein model of sufficient quality [36], [37]. We found mouse P-gp (PDB ID: 3G5U, 3G60 and 3G61), and Sav1866 (prokaryotic transport pump) to have the highest sequence identity and query coverage among all the proteins available in the PDB database, and chose them as a template to generate an open conformation ABCC6 homology model. BLAST result shows that ABCC6 shares a 26% sequence identity and 57% query coverage with mouse P-glycoprotein, and 27% identity and 58% coverage with Sav1866. Though within the ABCC transporter family ABCC6 has highest sequence homology with ABCC1 (44.045%), no PDB file of ABCC1 is listed in the PDB database. However, De Gorter et al. (2008) published an ABCC1 closed conformation model [20]; comprehensive literature mining revealed this to be the only available template which we used to generate a closed conformation ABCC6 homology model. ABCC6 shows 43.97% sequence identity and a 90.57% query coverage with the human ABCC1 model. Generally, native state or an open conformation state is a more stable form of a protein and more eligible for substrates to bind. Thus, to investigate potential substrate binding sites of a protein, use of an open conformation model is first choice. Conformational change of the closed conformation model during binding and release of the substrate is a common phenomenon in ATP-dependent transporters. Moreover, a closed conformation model expected to show low affinity or a higher binding energy to the substrate compared to an open conformation model. For these reasons, we selected P-gp as template in our principle docking ABCC6 open conformation model. An additional advantage of P-gp is the presence of complex ligands in inward facing conformation, which can provide information about the substrate binding sites (SBS) in ABCC6. It has been previously shown that TMD_0_ and L_0_ are responsible for the membrane localization as N-terminal truncated ABCC6 mutants missing both transmembrane (TMD_0_) and cytoplasmic linker (L_0_) domains were found to be absent from the plasma membrane [3]. There is however no evidence of TMD_0_ being related to substrate binding. Rather, the reported experiments indicate that the core region of ABCC6 is more likely to be involved in substrate recognition and binding. Moreover, as the counterparts of TMD_0_ and L_0_ are unavailable in databases, those external domains were excluded from the N-terminal region (1–301 amino acids) of the ABCC6 amino acid sequence. Contrary, our models contain the recently reported conserved C-terminal PDZ-like sequence- facilitating protein-protein interactions by acting as scaffold, regulating protein activity, protein stability, and protein mobility in the membrane - which was found to be critical for the regulation of trafficking and membrane localization of ABCC6. Removal of or mutation in this sequence results in decreased protein expression and increased degradation of ABCC6 [38]. We also have generated an open conformation 3D homology model including all 1503 amino acid residues by using P-gp as a template, but failed to overcome the quality threshold (Figure S2) as discussed below.

**Figure 1:**
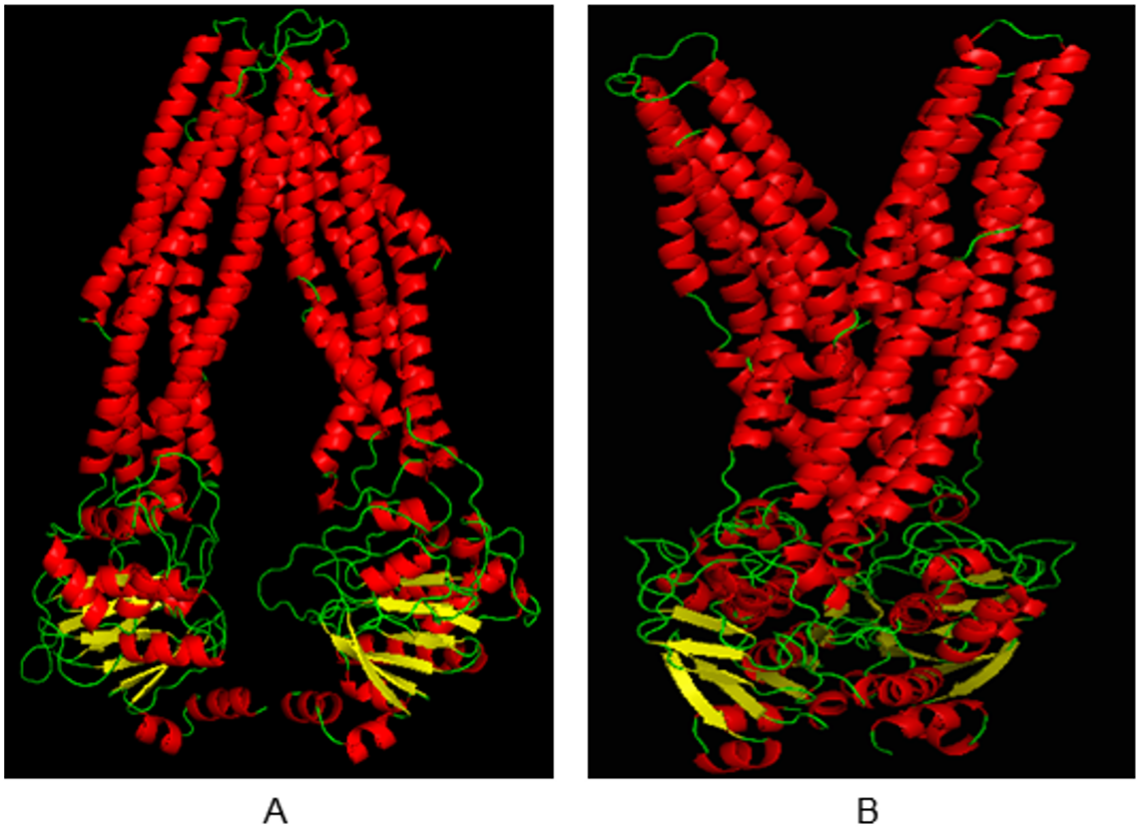
Human ABCC6 protein homology models, visualized in PyMOL. A) Ribbon representation of the model in wide open conformation. B) Ribbon representation of the model in closed conformation. Helix and Sheet regions are shown in red and yellow respectively, whereas the green color represents the loop regions. Similarly, green spheres represent those amino acid residues that fall into the disallowed region of the Ramachandran plot.

To prevent generation of low quality models due to low target-template sequence identity, we used three different Metaservers (I-Tasser, GeneSilico, and Pcons) and the MODELLER program, and generated four initial models for each server and program. Initially, MODELLER used 3G5U, 3G60 and 3G61 as templates and generated a good open conformation model. I-TASSER also resulted in a reliable model with a C-value of −0.31 (the C-value indicates the confidence score calculated by I-TASSER for the predicted model and ranges from −5 to 2) and a TM score of 0.67 (the TM score measures the structural similarity between two structures; a TM score >0.5 indicates a model of correct topology) (Figure S3). Then, by using these models as templates, the final open conformation model was generated by using MODELLER which generate a high quality model with a DOPE score of −90805.80 (Figure 1). In PyMOL, two chains (chain A and chain B) were detected in the ABCC6 structure. Chain A had been constructed by the amino acids from 302 to 856 and chain B from 857 to 1503. Furthermore, the loop regions less than 12 residues of the final model (560–565, 973–979, 1189–1200, 1221–1225) were remodeled using the ModLoop server, as it is very difficult to model loops having more than 12 residues [39]. For the closed conformation model (Figure 1), only MODELLER program (obtained DOPE score of −131757.60) and the counterparts of the template were used to generate the model.

### Validation of 3D homology models

Validation of the stereo-chemical properties of the final models was done using three servers: Rampage, Errat and ProQ. Stereo-chemical quality results for the open conformation ABCC6 model obtained by Rampage (Figure S4A) shows that 96.6% residues are in the favored region, 3.0% residues are in the allowed region and 0.4% residues are in the outliner region. In the closed conformation ABCC6 model, Rampage showed that 92% of the amino acids residues are plotted in the favored region, 7.1% residues are in the allowed region and 0.3% residues are in outliner region (Figure S4B). Third, an open conformation full-length ABCC6 model (Figure S2A) containing all amino acid residues (thus including the disordered region) did not pass the quality threshold as only 77.5% of amino acid residues were plotted in the favored region (Figure S2B). The residues in the outliner or disallowed region belong to either the disordered region or the NBD domain and are thus less likely to play a role in substrate binding. Model validation by the ERRAT web server (http://nihserver.mbi.ucla.edu/ERRATv2) showed an overall quality factor of 94.858% and 83.34% in the open and closed conformation ABCC6 model respectively (Figure S4C, S4D), where a value above 90% usually indicates a high quality model [21]. The results from the ProQ server demonstrates a ProQ LG score of 4.717 and 4.412, and a ProQ MaxSub score of 0.309 and 0.279 in the open and closed conformation model respectively. A proQ LG score of >0.3 indicates good quality of the model and a ProQ MaxSub score of >0.1 indicates ‘correct’ [24]. SuperPose showed the superimposed images of template 3G5U and I-TASSER-model with the final model of ABCC6 separately (Figure S5A and S5B respectively). It depicted the 3D alignment of template and target protein. In SuperPose, we found an identity and similarity of 21.9% and 38.5% respectively for the 3G5U-open conformation ABCC6 structural alignment. For open conformation-ABCC6-I-TASSER, both the identity and similarity was 79.9%. SuperPose shows 45.1% of identity and 64% of similarity for the ABCC1-closed conformation ABCC6 model, and of 21.7% of identity and of 40.7% similarity for the P-gp-based full-length ABCC6 model. Therefore, both the 3G5U and I-TASSER-model had good coverage of length and structure, where the spatial organization of the target protein is much closer to the I-TASSER-model. In addition, sequence alignment of template and open conformation ABCC6 (Figure S6), shows the closeness between these two models. Thus, overall quality validation revealed that the open conformation ABCC6 homology model generated using P-gp as a template is the most accurate model to perform molecular docking analysis.

### Analysis of topology and secondary structure, and prediction of protein disordered region

According to previous studies, the membrane topology model of human ABCC6 is composed of 17 transmembrane helices with the topology arrangement of TMD0-L0-TMD1-ABC1-L-TMD2-ABC2, two nucleotide-binding domains (NBD1 and NBD2) and two Walker motifs Walker-A and Walker-B [3]. The TMD0 is formed by five membrane spanning helices whereas the TMD1 and TMD2 domains are formed by 6 membrane helices each [3], [40]. The predicted result found by the PONGO server (presented only for the open conformation ABCC6 model in Figure S7) in our study shows a similar and consistent conformation. This result is also supported by the TMHMM server result where it showed a plot of posterior probabilities (0 to 1) of inside, outside and TM helix. At the top of the plot (between 1 and 1.2) the N-best predictions are shown. TMHMM showed two major spots of transmembrane helices from 302–600 and 900–1225 as expected. In TMHMM, the first and second portion present 6 and 4 membrane helices respectively, while PONGO predicts 6 membrane helices in both portions.

The intrinsically disordered/unstructured region of a protein is the region which does not have a well-defined three dimensional structure and can exhibit a large number of conformations over time [41]. The region between amino acid residues 856 to 943 in our study was found to be disordered and less likely to form a stable structure for the ABCC6 protein (Figure S8). Indeed, the model including this region did not fulfil the quality criteria. This disordered region was removed from the ABCC6 sequence in the open and closed confirmation model, and thus ignored for model generation and blind docking analysis.

### Blind docking analysis for the prediction of substrate binding sites

Since the substrate binding sites of the ABCC6 transporter are yet to be defined, we used a blind docking approach by using Autodock tools for the 8 compounds that were reported to be transported in vitro by ABCC6 as reference substrates to our models. In addition, because of the suggestion of involvement of vitamin K in the PXE pathophysiology, we also performed blind docking for the three known vitamin K isoforms: VK1, VK2 and VK3. The latter has already been shown not be transported by ABCC6 and was thus used as a negative control [30].

In the blind docking approach, by using the grid volume that covers the whole transmembrane region of the protein for the 10 compounds (8 reported substrates, VK1 and VK2) distributed over the open conformation homology model the potent binding areas were predicted. Further, a refined docking for specific areas was carried out to optimize the results. This refined docking approach resulted in 20 different binding conformations for each compound. These conformations were clustered together with RMSD (root mean square deviation) <4 A°. The lowest binding energy conformation from the largest cluster was chosen.

**Figure 2:**
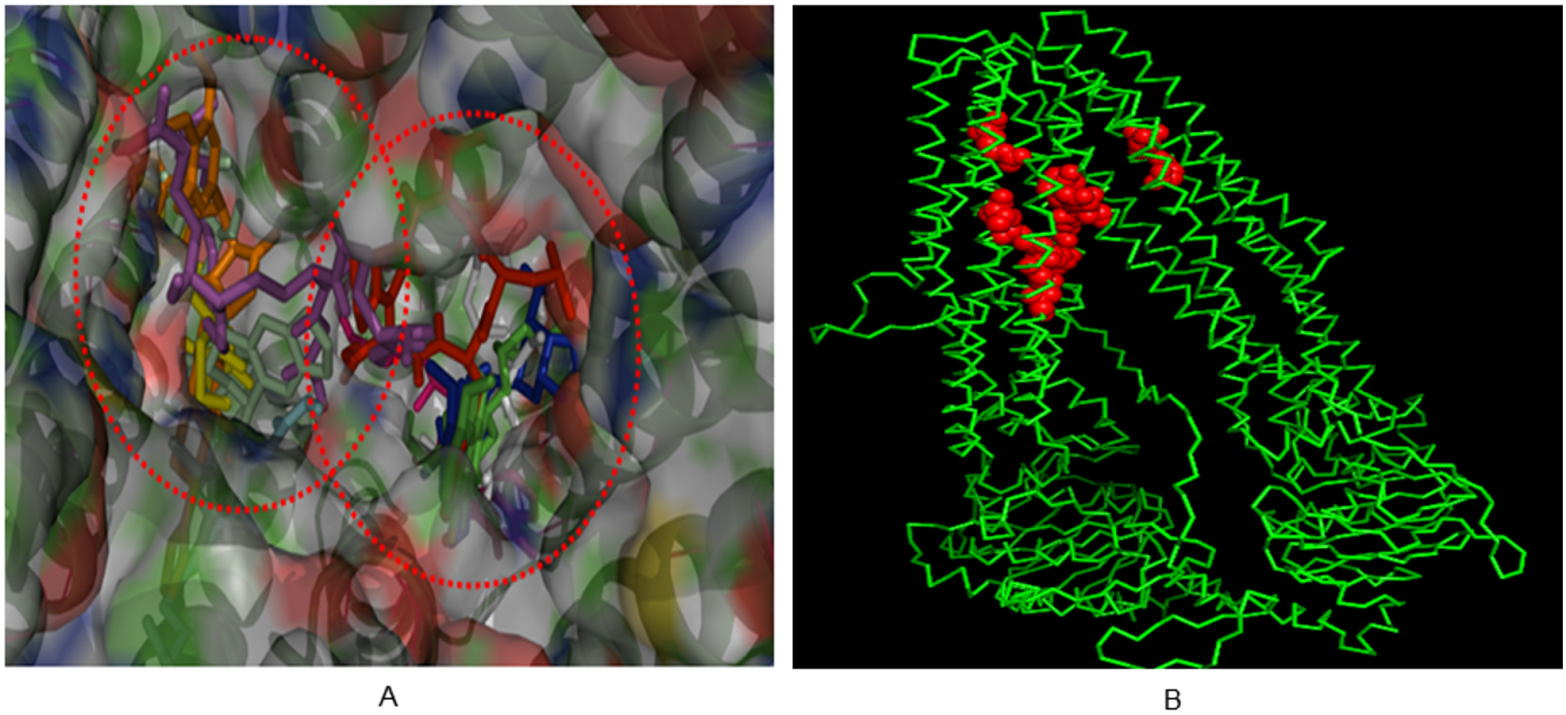
Docking of ten reference compounds revealed the presence of two substrate binding sites in the ABCC6 open conformation model. A) Intracellular view of the docked reference molecules showed that all the molecules are clustered in two substrate binding sites (SBS) of the ABCC6 homology model (indicated by two red dotted circles). In addition, intersections of the reference molecule in the binding sites revealed the overlap and flexibility of the binding sites. B) Location of SBS in the ABCC6 open conformation structure represented in PyMOL. The 3D structure of ABCC6 is green and the major amino acids involved in SBS are highlighted in red.

From the blind docking and subsequent refined docking, the 10 reference compounds were found to bind in two specific hotspot areas of the open conformation ABCC6 model, which may indicate the presence of two substrate binding sites (SBS; Figure 2). Structure analysis revealed that both chain-A (302–856AA) and chain-B (857–1503AA) are involved in the conformation of SBS-1 (substantial amino acids: Ser470, Asn474, Gln540, Thr543, Asn575, Asn578, Lys579, Arg964, Gln1217, Trp1218, Arg1221) and involved in SBS-2 (substantial amino acids: Glu1176, Asn1180, Thr1213). Moreover, docking result shows that SBS-1 is formed by TM6, TM9, TM10, TM11, TM12, TM13, TM14 and TM17, whereas SBS-2 is formed by TM6, TM7, TM15, TM16 and TM17. The interactions of reference compounds and SBS are enlisted in Table 1.

**Table 1:** Docking analysis of 10 reference compounds against human ABCC6 model in open wide conformation along with the binding free energy value (Kcal/mol) form Autodock and Autodock Vina.

Compound Name	Autodock Docking Energy (Kcal/mol)		Autodock Vina Energy (Kcal/mol)		Predicted inhibitory constant	Amino acid residue involvedOpen (ATP free state)		
	Open Conf.	Closed Conf.	Open Conf.	Closed conf.		H-Donor	H-acceptor	H bond distance (Å)
BQ-123	−8.42	−7.11	−10.9	−7.9	677.38 nM	A:LYS579:HZ2	N:UNK1:O	1.73718
						A:LYS579:HZ3	N:UNK1:O	2.26551
						B:GLN1217:HE22	N:UNK1:O	1.75979
Teniposide	−9.81	−6.40	−9.3	−7.3	64.25 nM	A:GLN540:HE21	N:UNK1:O	2.37559
						A:GLN540:HE22	N:UNK1:O	2.16689
						A:THR543:HG1	N:UNK1:O	1.93108
Etoposide	−7.56	−6.04	−9.0	−7.3	2.85 µM	A:THR543:HG1	N:UNK1:O	2.13517
						B:ARG1221:HH12	N:UNK1:O	1.78112
						N:UNK1:H	A:ASN578:O	2.23698
Doxorubicin	−6.03	−4.67	−9.2	−6.8	38.17µM	N:UNK1:H	B:GLU1176:O	2.4355
						N:UNK1:H	B:GLU1176:OE1	1.91985
						N:UNK1:H	B:ASN1180:OD1	2.20217
						N:UNK1:H	B:THR1213:OG1	2.25081
NEM-GS	−5.39	−3.42	−7.7	−5.4	111.26 µM	A:ASN575:HD22	N:UNK1:O	2.18442
						A:LYS579:HZ1	N:UNK1:O	2.13871
						A:LYS579:HZ3	N:UNK1:O	1.80004
						B:GLN1217:HE22	N:UNK1:O	2.3928
						B:TRP1218:HE1	N:UNK1:O	2.18593
						N:UNK1:H	B:GLN1217:OE1	2.1851
S-2(2, 4-dinitrophenyl) glutathione	−8.32	−3.20	−6.8	−4.6	384.99 µM	A:ASN575:HD22	N:UNK1:O	1.93551
						A:LYS579:HZ1	N:UNK1:O	2.11314
						A:LYS579:HZ1	N:UNK1:O	2.36311
						B:ARG964:HH12	N:UNK1:O	2.16605
						B:ARG1221:HH11	N:UNK1:O	2.11578
						B:ARG1221:HH11	N:UNK1:O	2.21185
						N:UNK1:H	B:GLN1217:OE1	2.15382
						N:UNK1:H	B:GLN1217:OE1	2.12873
Vitamin K1	−8.2	−5.78	−7.2	−5.7	972.49nM	No hydrogen bonding		
Vitamin K2	−7.72	−6.59	−9.5	−6.2	4.72 µM	No hydrogen bonding		
Daunorubicin	−6.37	−6.06	−8.5	−6.6	281.39 nM	N:UNK1:H	B:GLU1176:OE1	1.99061
						N:UNK1:H	B:THR1213:OG1	2.40524
Leukotriene C(4)	−4.03	−2.38	−7.3	−5.3	1.1 mM	A:ASN474:HD21	N:UNK1:O	2.14932
						A:ASN474:HD22	N:UNK1:O	2.36946
						A:LYS579:HZ3	N:UNK1:O	2.1131
						B:GLN1217:HE22	N:UNK1:O	2.15727
						N:UNK1:H -	A:SER470:OG	2.25413
						N:UNK1:H -	B:GLN1217:OE1	2.36306

The residues that contribute as H-bond donor or H-bond acceptor during interaction with substrate molecules are also shown along with their relative bond distance. SBS: substrate binding site; Conf.: Conformation.

**Figure 3:**
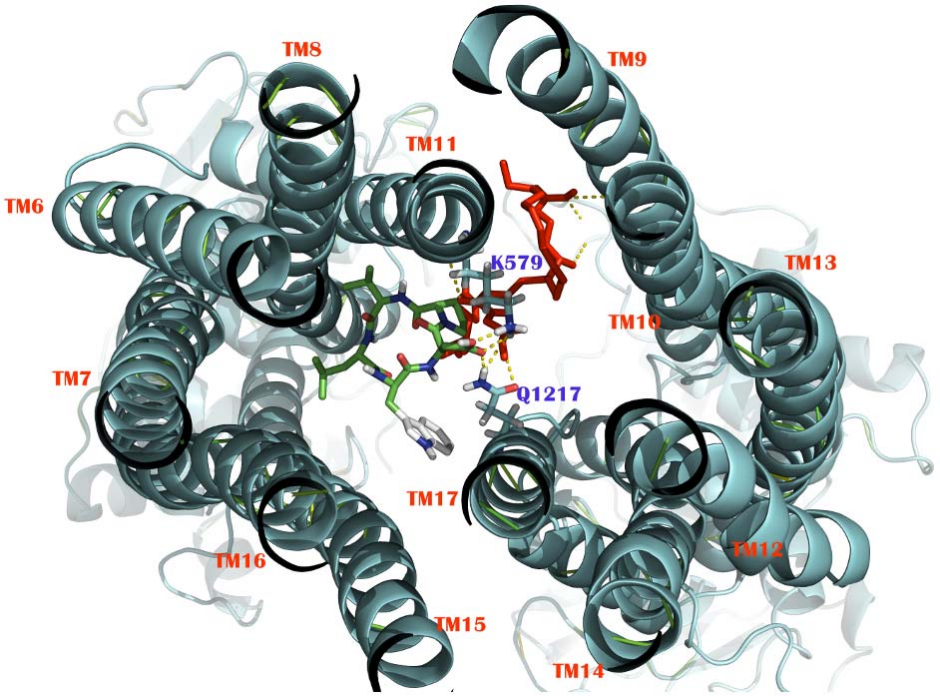
Extracellular face view of an arrangement of transmembrane (TM) regions at the proposed binding sites. Docking poses of substrate BQ-123 (green) and leukotriene C4 (red) into two different predicted substrate binding sites in the human open conformation ABCC6 homology model overlap partially with each other, sharing common residues Lys579 and Gln1217 by forming hydrogen bonding. The 12 transmembrane domains are numbered TM6 to TM17 (red).

We noted that some ligands that bind in these predicted binding sites also partly overlap with each other (Figure 2A and Figure 3), thus resembling a large flexible cavity instead of two separate binding pockets. The presence of such a large flexible substrate binding cavity has been shown for several ABC transporters (e.g. P-gp, ABCC1) and allows the possibility of a wide range of substrates [42], [43].

On the other hand, refined docking of the 10 reference compounds shows that all the compounds bind in a specific area of the closed conformation ABCC6 model. Structure analysis revealed only Chain-A (302–856AA) to be involved in the substrate binding site conformation (substantial amino acids are Ser470, Gln540, Thr543, Asn575, Asn578, Lys579), which revealed >55% amino acid residues similarity to SBS-1 in the open conformation ABCC6 homology model. Taking into account the closed conformation refined docking result, this is suggestive for ABCC6 to have a single big flexible binding pocket. However, we need to take into account that the closed conformation model result may be less reliable and definite conclusion at this point are therefore not possible.

**Figure 4:**
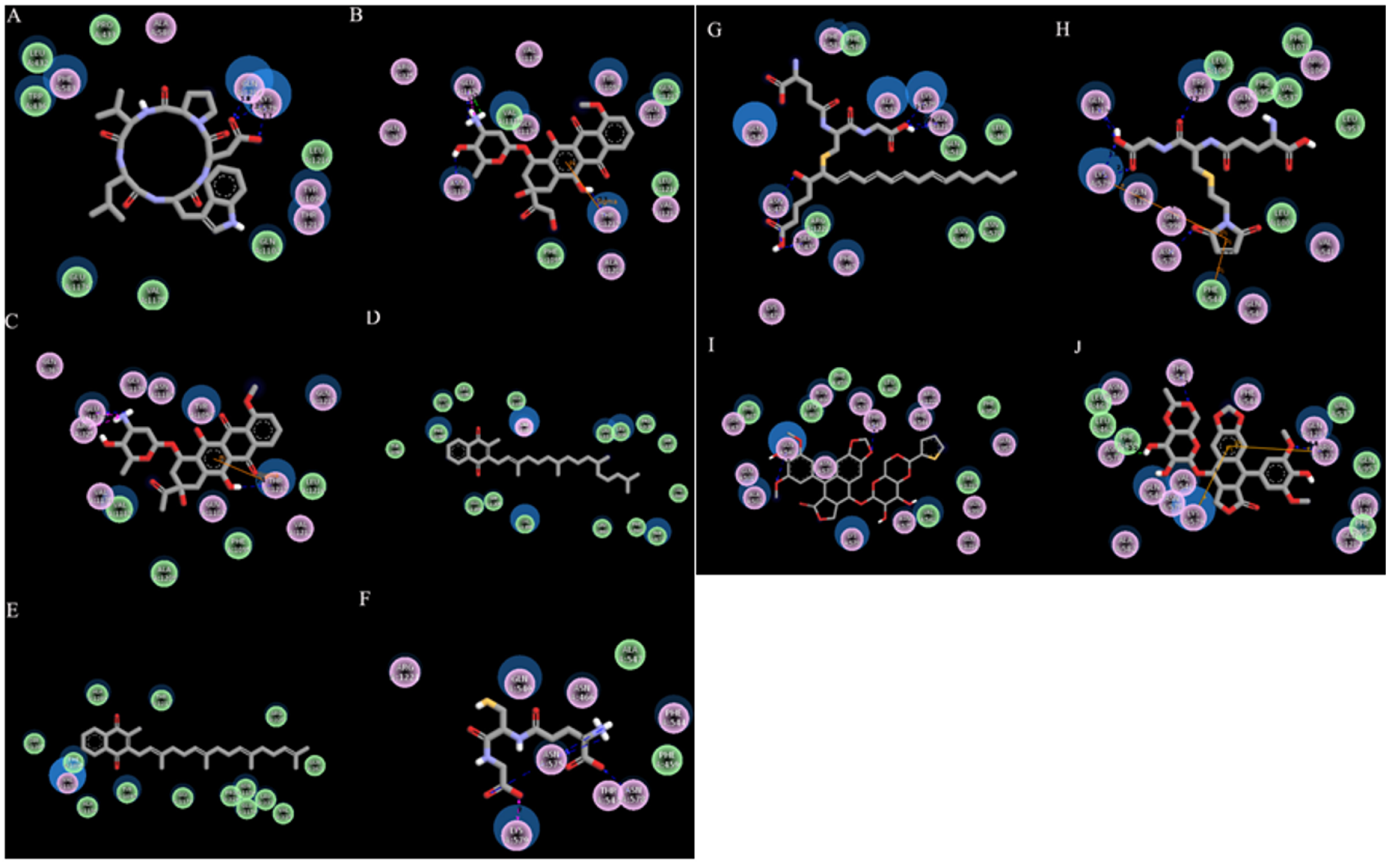
Two-dimensional representations of the 10 reference compounds, showing H-bond and hydrophobic interactions within substrate binding sites of ABCC6 open conformation. (A) BQ-123, (B) Doxorubicin, (C) Daunorubicin, (D) Vitamin K1, (E) vitamin K2, (F) S-2(2, 4-dinitrophenyl) glutathione, (G) Leukotriene C4, (H) NEM-GS, (I) Teniposide, and (J) Etoposide are shown respectively. Residues involved in Van der Waals interaction are represented by green disks, residues involved in polar interaction or hydrogen bonding are represented by pink disks and solvent accessible surfaces of the interacting residues are represented by a blue halo around the residues.

Remarkably, average docking energy of Autodock and Autodock Vina in both models showed highest binding affinity for compound BQ-123 followed by teniposide. BQ-123 was seen to interact with the active site residue Lys579 by forming two hydrogen bonds with bond distance of 1.73A° and 2.26A° and single hydrogen bond with residue Gln1217 with bond distance of 1.75A°. Teniposide was found to interact with residue Gln540 by two hydrogen bonds with bond distance of 2.375A° and 2.166A° and single hydrogen bond with residue Thr543 with bond distance of 1.93A°. In contrast, VK3 shows low binding affinity and higher binding energy (than the average of 10 reference compounds). Moreover, this binding energy was generated for an outer face site in the open conformation ABCC6 model (Figure S9). Its low affinity and lack of specificity for a binding site is consistent with VK3 not being transported by ABCC6. The final docking result of the 10 compounds along with corresponding binding energy for both models is illustrated in Table 1 and the representative binding mode is shown in Figure 4. Residues involved in hydrophobic interactions with corresponding substrate molecules are shown in Table S2.

Furthermore, multiple sequence alignment with the two most closely related members of the human ABC family, ABCC1 and ABCC2 shows that most of the residues in the predicted binding regions from ABCC6 are found to be conserved with those residues of ABCC1 or ABCC2 that are reported to play significant role in substrate binding (Figure S10) [44], [45], [46], [47]. Residues such as Trp1218 contributing in hydrogen bonding and hydrophobic interactions between ABCC6 and its substrates, was found conserved. Asn575, Asn578, Lys579, Thr1213 and Arg1221 residues are conserved among ABCC6 paralogs, found in the substrate binding pockets of other ABC proteins, and have been described to be efficient for substrate specificity and transport function [45], [47], [48], [49], [50]. As shown in Table S2, the majority of residues that contribute in the interactions with substrate molecules are present in TM 9, 10, 11, 16 and 17. This is consistent with a number of observations suggesting that ABCC1, ABCB1 and other related ABC proteins possess multiple substrate binding sites and that the transmembrane domains such as TM9, 10, 11, 16 and 17 play a significant role in substrate recognition and binding [42], [51], [52].

Evaluation of the mutations which affect the amino acids involved in SBS revealed that, among 288 known unique mutations (www.ncbi.nlm.nih.gov/lovd/), only 16 mutations have been found to be involved in SBS-1, and 4 mutations have been found in SBS-2 of the open conformation model (Table 2). This result may signify that few ABCC6 mutations cause disruption of substrate binding to ABCC6, but that most disease causing mutations of ABCC6 are probably involved in Nucleotide Binding Domain (NBD) or other portions of the protein that are important for protein localization, so that it causes the inactivation or mis-localization of ABCC6. This was recently also suggested by Xue et al. [38].

**Table 2:** List of ABCC6 mutations reported in the predicted binding sites of ABCC6 open conformation model.

Substrate Binding Site-1						
	AA involved	Variant	Protein	Variant type	Exon	Protein change
**Chain A**	L463	1388T>A	L463H	Missense	11	p.(Leu463His)
	N466	1396A>T	N466Y	Missense	11	p. (Asn466Tyr)
	F568	1703C>T	F568S	Missense	13	p.(Phe568Ser)
	F568	1703T>C	F568S	Missense	13	p.(Phe568Ser)
	L946	2836C>A	L946I	Missense	22	p.(Leu946Ile)
	L953	2868T>A	L953H	Missense	22	p.(Leu953His)
	R964	2891G>C	R964P	Missense	22	p.(Arg964Pro)
	998	998+2delT		Splice	8i	
	998	998+2_998+3delTG		Splice	8i	
**Chain B**	D1006	3307–1006_3882+1582del		Del	23i_25i	
	D1056	3168C>A	D1056E	Missense	23	p.(Asp1056Glu)
	R1064	3190C>T	R1064W	Missense	23	p.(Arg1064Trp)
	G1203	3608G>A	G1203D	Missense	25	p.(Gly1203Asp)
	R1221	3661C>T	R1221C	Missense	26	p.(Arg1221Cys)
	R1221	3662G>A	R1221H	Missense	26	p.(Arg1221His)
	W1223	3668G>A	W1223X	Nonsense	26	p.(Trp1223*)
**Substrate Binding Site-2**						
**Chain A**	Q363	1087C>T	Q363X	Nonsense	9	p.(Gln363*)
	Q363	1088_1120del33	Q363_R374del	Del	9	p.(Gln363_Arg374)
	Q363	1087C>T	Q363X	Nonsense	9	p.(Gln363*)
	Q363	1088_1120del33	Q363_R374del	Del	9	p.(Gln363_Arg374)

AA: amino acid; Splice: splice site mutation; Del: deletion.

### Electrostatic Potential Surfaces (EPS)

**Figure 5:**
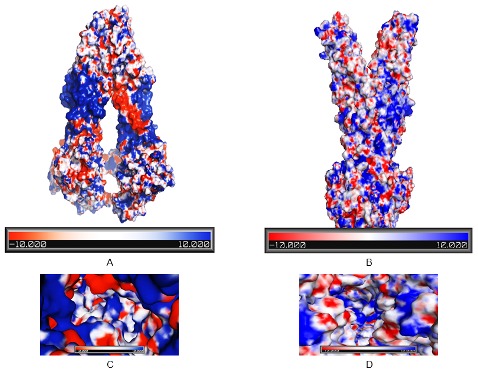
Electrostatic potential surface of the human ABCC6 model with the predicted substrate binding sites (boxed). (A) EPS for open conformation ABCC6 model, (B) EPS for closed conformation ABCC6 model. (C, D) EPD of substrate binding site(s) in open and closed conformation respectively. Electronegative potential scale of −10 kcal/mol to +10 kcal/mol is shown. Blue, red and white colors represent positively, negatively and neutrally charged areas respectively.

EPS of the ligand recognition area in the ABC transporters is of particular interest, since EPS can elucidate the substrate differentiation between these transporters [53]. For this reason, the EPS value of our models was calculated using the PBEQ solver program. Analysis of the electrostatic charge distribution in our models revealed variations in surface electrostatic at different cavities in the ABCC6 protein (Figure 5). Our study shows that the predicted substrate binding site(s) of ABCC6 model exhibit an overall positive (or polar) with neutral (or hydrophobic) and large negative area (Figure 5). Most of the positively charged residues (in blue color) were found in the cytoplasmic part of the protein, whereas mostly neutral (or hydrophobic; in white color) with some spots of positively and negatively (in red color) charged residues were found at the transmembrane region of the protein surface. The presence of hydrophobic pockets with spots of negatively and positively charged residues indicate the role of these residues in electrostatic interaction with specific substrates [54]. Moreover, this also suggest that the negatively charged substrates such as S-2(2, 4-dinitrophenyl glutathione), Leukotriene (LTC4) and BQ-123 might interact electrostatically with positively charged residues within two predicted binding regions whereas the positively charged and neutral hydrophobic substrates such as Doxorubicin, Daunorubicin, Etoposide, Teniposide, vitamin K1 and vitamin K2 might interact electrostatically with negatively charged residues of the ABCC6 open conformation model. Since the majority of multidrug transporters transport drugs with a positive charge, it is reasonable to assume the involvement of negatively charged intra-membranous acidic residues in substrate binding [54]. The role of these residues in substrate recognition has already been reported in number of MDR proteins such as the E.coli MdfA protein [55] and in murine MRP1, where a single ‘Glu’ residue at the TM15 region was found to have a role not only in transporting cationic anthracyclines but was also important in transporting conjugated organic anions, such as LTC4 [56].

### Virtual screening of Serum Metabolome Database

The virtual screening of 4,651 compounds from HSMD in the ABCC6 open conformation model using mean binding energy −8.5 Kcal/mol for Autodock Vina and −7.2 Kcal/mol for Autodock from blind docking as a cut-off threshold value (Table S1) resulted in the selection of the top 50 compounds (1.075% of the total compounds in HSMD) (Table 3). Most of the compounds found in this virtual screening are lipid compounds and biliary secretions. The compounds in the list can be categorized into the following five classes: lipids (30 compounds), biliary secretions (10 compounds), aromatic hetero-polycyclic compounds (5 compounds), amino acids, peptides and analogs (3 compounds), nucleosides, nucleotides and analogs (1 compound), and homogeneous non-metal (1 compound). Virtual screening and docking of these top 50 compounds in the closed conformation model showed lower affinity (higher binding energy) to the compounds than in the open conformation model. The binding energy is consistently higher (for every compound) in closed conformation than the open conformation. The average difference of binding energy (with the compounds) of open and closed state model ranges from 1.5 Kcal/mol to 3.0 Kcal/mol, that would be the possible substrate-release energy due to conformational change of the protein. This explains the substrate binding in a native or open conformation structure of ABCC6 and then it goes to the closed state after ATP binding, and releases the substrate by lowering the binding-affinity.

**Table 3:** List of 50 compounds obtained after virtual screening from the Human Serum Metabolome Database (HSMD) containing 4,651 compounds (and their involvement in calcification related mechanism) in open conformation model, by using the docked reference substrate molecules’ mean binding energy (-8.5 Kcal/mol found from Autodock-Vina) as cut-off value.

Sl. No.	Accession No.	Autodock vina docking energy (Kcal/mol)		Autodock docking energy (Kcal/mol)		Common name	Super-class	Relevance for calcification
		Open Conf.	Closed Conf.	Open Conf.	Closed Conf.			
1	HMDB01438	-10	−7.7	−10	−5.82	25-Hydroxyvitamin D2	Lipid	Low 25-hydroxyvitamin D is a strong risk indicator of vascular calcification [60].
2	HMDB00878	−9.8	−7.6	−8.82	−6.9	Ergosterol	Lipid	Low in diet causes bone calcification [61].
3	HMDB11181	−9.6	−7.4	−9.18	-6.64	Brassicasterol	Lipid	NA
4	HMDB01032	−9.5	−6.9	−8.65	−6.5	DHEA sulfate	Lipid	Significantly lower in aortic calcification [62].
5	HMDB01980	−9.4	−7.8	−5.7	−2	Vasopressin	Amino Acids, Peptides, and Analogues	Stimulates both Na^+^ dependent Pi transport and mineralization in VSMCs [63].
6	HMDB06327	−9.4	−7.3	−8.47	−4.73	Alpha-Tocotrienol	Lipid	Deficiency impairs bone calcium homeostasis [64].
7	HMDB00722	−9.3	−7.6	−9.67	−6.62	Lithocholyltaurine	Lipid (Bile acid)	Intracellular Ca2+ concentration homeostasis [65].
8	HMDB01045	−9.3	−7.2	−5.65	−2.5	Enkephalin L	Amino Acids, Peptides, and Analogues	NA
9	HMDB11628	−9.3	−7.9	−9.88	−6.86	Glycyrrhetinic acid	Lipid	Can reverse PTH-induced ECM mineralization [66].
10	HMDB01830	−9.2	−7.2	−8.2	−6.6	Progesterone	Lipid	Rare cause of tendon calcification [67].
11	HMDB00032	−9.1	−7.8	−8.96	−6.6	7-Dehydrocholesterol	Lipid	High vitamin D diet mice have phenotypic features of premature aging with ectopic calcification [68].
12	HMDB00241	−9.1	−7.6	−10.79	−6.22	Protoporphyrin IX	Aromatic Heteropolycyclic Compounds	Upregulation causes increased mineralization [69].
13	HMDB00760	−9.1	−7.5	−8.78	−6.33	Hyocholic acid	Lipid (Bile acid)	Candidate for anti-atherosclerotic drug therapy [70].
14	HMDB00374	−9	−7.3	−8.09	−6.1	17-Hydroxyprogesterone	Lipid	NA
15	HMDB00896	−9	−7.7	−9.52	−6.1	Taurodeoxycholic acid	Lipid (Bile acid)	NA
16	HMDB00907	−9	−7.2	−8.27	−6.78	Sulfolithocholic acid	Lipid (Bile acid)	NA
17	HMDB01425	−9	−7.8	−8.95	−5.9	Estrone sulfate	Lipid	NA
18	HMDB01993	−9	−7.3	−8.93	−5.69	7a-Hydroxy-cholestene-3-one	Lipid (Metabolite in bile acid synthesis)	NA
19	HMDB10335	−9	−7.5	−9.4	−4.7	Estriol-3-glucuronide	Lipid	Protects cardiovascular system by reducing oxidative stress [71].
20	HMDB00121	−8.9	−7.4	−6.6	−4	Folic acid	Lipid	Rare cause of metastatic calcification [72].
21	HMDB00138	−8.9	−7.8	−7.89	−6.65	Glycocholic acid	Lipid (Bile acid)	NA
22	HMDB00761	−8.9	−7.8	−9.12	−6.31	Lithocholic acid	Lipid (Bile acid)	Plays role in bone repair/regeneration by aiding matrix calcification at implant sites [73].
23	HMDB00917	−8.9	−7.5	−7.95	−4.77	Ursocholic acid	Lipid (Bile acid)	Induces apoptosis [74].
24	HMDB00946	−8.9	−7.2	−8.8	−6.1	Ursodeoxycholic acid	Lipid (Bile acid)	Prevents hepatocellular apoptosis [75].
25	HMDB01926	−8.9	−7.8	−7.98	−6.1	17a-Ethynylestradiol	Lipid	Acts as anti-atherogenic signal and reduces aortic calcification [76].
26	HMDB10356	−8.9	−7.5	−7.49	−5	Estriol 3-sulfate 16-glucuronide	Lipid	NA
27	HMDB00774	−8.8	−7.7	−9.36	−6.6	Pregnenolone sulfate	Lipid	Can be used as therapeutic vascular modulator [77].
28	HMDB01056	−8.8	−7.4	−6.78	−4	Dihydrofolic acid	Aromatic Heteropolycyclic Compounds	NA
29	HMDB01846	−8.8	−7.7	−7.31	−3	Tetrahydrofolic acid	Aromatic Heteropolycyclic Compounds	Lower concentration in progressive intracranial calcification [78], [79].
30	HMDB01449	−8.8	−7.7	−8.54	−6.34	Allopregnanolone	Lipid	NA
31	HMDB01562	−8.8	−7.1	−7.85	−4	N5-Formyl-THF	Aromatic Heteropolycyclic Compounds	NA
32	HMDB01893	−8.8	−6.4	−8.11	−4	Alpha-Tocopherol	Lipid	Supplementation causes significant reduction of villus calcification [80].
33	HMDB00494	−8.7	−6.5	−8.55	−6.1	Stigmastanol	Lipid	NA
34	HMDB00570	−8.7	−7.8	−9.25	4.71	Coproporphyrin III	Aromatic Heteropolycyclic Compounds	NA
35	HMDB01903	−8.7	−7.1	−8.56	−4.9	Calcitriol	Lipid	Potential for cardiovascular protection without the risk of inducing intracellular calcification [81].
36	HMDB06119	−8.7	−6.9	−9.25	−5.7	7b-Hydroxycholesterol	Lipid	Increased levels correlate with increased risk of cardiovascular diseases including atherosclerosis [82].
37	HMDB06552	−8.7	−6.9	−7.5	−5.11	Aflatoxin B1	Aromatic Heteropolycyclic Compounds	Interaction with Vitamin D3 plays important role in bone calcification [83]
38	HMDB06759	−8.7	−7.1	−8.19	−6.75	3a-Hydroxy-5b-pregnane-20-one	Lipid	NA
39	HMDB00492	−8.6	−7	−7.32	−5.3	Chlorine	Homogeneous Non-metal Compound	NA
40	HMDB00546	−8.6	−6.9	−8.4	−6.22	Epietiocholanolone	Lipid	NA
41	HMDB00852	−8.6	−7.6	−8.08	−5.47	Beta-Sitosterol	Lipid	Supplementation in diet inhibits atherosclerosis in rabbits [84]
42	HMDB00876	−8.6	−7.6	−9.08	−5.67	Vitamin D3	Lipid	Induces significant intracellular calcification [85]
43	HMDB05015	−8.6	−6.4	−7.29	−4.79	Gabapentin	Amino Acids, Peptides, and Analogues	Reduces calcium influx in respiratory muscles [86].
44	HMDB00326	−8.5	−7.2	−9.14	−5.68	1b,3a,12a-Trihydroxy-5b-cholanoic acid	Lipid (Bile acid)	NA
45	HMDB00490	−8.5	−7	−8.04	−5.83	Etiocholanolone	Lipid	NA
46	HMDB00628	−8.5	−7.5	−8.45	−5.98	Epitestosterone	Lipid	Causes decreased bone density and calcium content [87]
47	HMDB00908	−8.5	−7.7	−10.71	−6.3	5alpha-Cholestano	Lipid	NA
48	HMDB00937	−8.5	−7.4	−9.45	−6.8	Stigmasterol	Lipid	Prevents calcification of the pineal gland and plays role in elimination of phosphorus and calcium [88].
49	HMDB01170	−8.5	−7.5	−8.47	−6.61	Lathosterol	Lipid	NA
50	HMDB10340	−8.5	−7.9	−7.02	−4.04	Retinyl beta-glucuronide	Lipid	NA

Validations of the best 50 compounds were done by docking in closed conformation to see the possible good affinity with the model. S.: serial number; Acc. No.: accession number; Kcal: Kilocalory, HMDB: human metabolome database; ABC: ATP-binding cassette transporter; VSMCs: vascular smooth muscle cells; ECM: extra cellular matrix; NA: not available.

**Figure 6:**
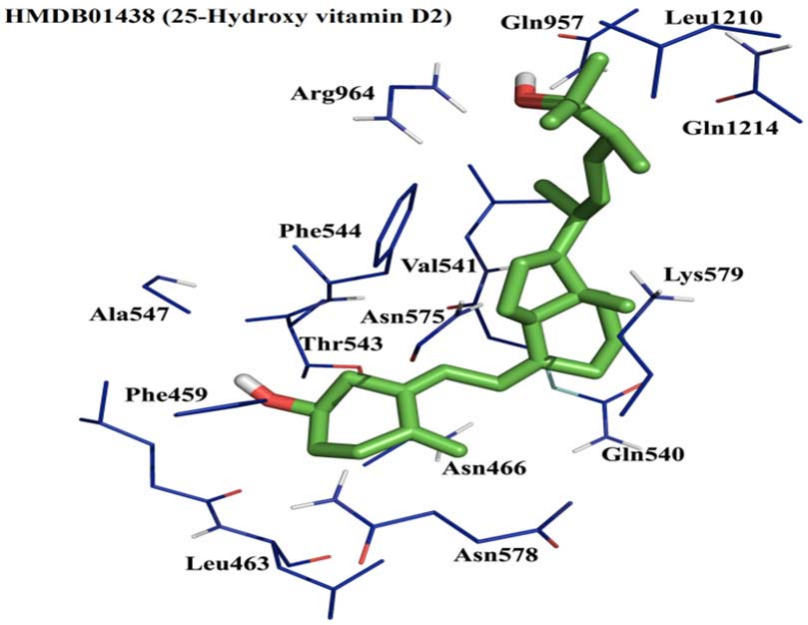
Predicted docking pose of the top scoring compounds from the Human Serum Metabolome Database (HSMD) in the open conformation ABCC6 homology model. Line representation (blue) of surrounding amino acids involved in the interaction with the respective compound (stick representation in green color). HMDB01438 (25-Hydroxy vitamin D2)-ABCC6 interaction in substrate binding site of ABCC6 generated a binding free energy of −10 Kcal/mol. Residues such as Thr543, Gln957, Arg964 are involved in the hydrogen bond interaction while the other surrounding amino acid residues are involved in hydrophobic interactions.

From the listed 50 compounds, several compounds are involved in vitamin D metabolism, disturbances of which can play a role in calcification [57], [58]. The highest affinity for SBS-1 with a binding free energy of −10 Kcal/mol, was found for 25-Hydroxyvitamin D2 (HMDB01438) in ABCC6 open conformation model. It forms 3 hydrogen bonds with residues Thr543, Gln957, Arg964 with bond distance of 2.1A°, 1.8A°, and 2.3A° respectively (shown in Figure 6). Besides vitamin D metabolites, several other compounds were found to be involved in in calcification and calcification related mechanisms (Table 3). Several of the listed compounds, such as HMDB01830, HMDB00760, HMDB00907, HMDB00121, HMDB00761, HMDB00917, HMDB00946, HMDB01926, HMDB01562, and HMDB00326, have previously been reported to be transported by other ABC transporter proteins [59]. Further in vitro and in vivo validation is necessary to evaluate whether the actual substrates of ABCC6 are among these compounds.

## Conclusion

The current study provides an insight of the 3D structure and predicted substrate binding sites of the ABCC6 transporter. Molecular docking of reference substrates predicted two binding sites or a large flexible binding pocket of the protein, which allowed searching novel substrates of ABCC6 by performing ligand-based virtual screening. We have identified compounds from HSMD which bind with high affinity to the predicted substrate binding sites of the ABCC6 protein. The amino-acid residues which are involved in the predicted binding sites are conserved among ABC transporters and were also found to reside in the binding sites of ABCC1 and ABCC2 (Figure S10). Though this can be seen as indirect support of the relevance of our findings, the main limitations of this study remain that it is based on in silico modeling and prediction. For the blind docking experiment with HSMD metabolites, we have so far only considered the 50 compounds with highest binding affinity. We should also consider the possibility that the substrate of ABCC6 is ranked lower than the top 50 of these compounds, that more than one of the top 50 compounds can be transported at minimal level by ABCC6 and that there may be a third substrate binding site for the main substrates of ABCC6. Further, we cannot exclude that the ABCC6 substrate has no direct relation to mineralization but is an intermediate in a regulatory process. Nevertheless, the results of this study provide opportunities to further analyze in vitro and in vivo compounds which may interact with ABCC6, with potential insights into the function of this transporter and subsequently the mechanisms underlying PXE.

## Supporting Information

Figure S1 **Two dimensional structure of the 8 reported in vitro substrates and 3 vitamin K isoforms.**
(TIF)

Figure S2 **Human ABCC6 protein homology model containing full-length amino acid residues.** (A) Ribbon representation of the model in wide open conformation. Helix and sheet regions are shown in red and yellow respectively, whereas the green color represents the loop regions. (B) Ramachandran plot showing distribution of amino acid residues in favored, allowed and disallowed region.
(TIF)

Figure S3 **TMHMM plot shows the probability of the presence of TM region in the sequence.** It shows the amino acid-wise probabilities for inside, outside and transmembrane region of the ABCC6 sequence. Probability ranges from 0 to 1. At the top of the plot, between 1 to 1.2, the best predictions have been shown as summary. Figure shows 6 TM regions at the first 300 aminoacids, and 4 TM regions from amino acid position 600 to 950.
(TIF)

Figure S4 **Stereo-chemical validation of the human ABCC6 protein models.** Ramachandran Plot generated by the RAMPAGE server shows that- (A) in the open conformation model 96.7% of residues are plotted in the favored region (dark cyan color), 3.0% of residues in the allowed region (cyan color) and 0.4% of residues in the outliner region (red dot), (B) in the closed conformation model 92% of the amino acids residues are plotted in the favored region, 7.1% residues are in the allowed region and 0.3% residues are in the outliner region. (C, D) ERRAT plot for the ABCC6 model as predicted by the ERRAT server. ERRAT showed that the model has an overall quality factor of 94.858% (C) and 83.345% (D) in the open and closed conformation respectively.
(TIF)

Figure S5 **The SuperPose calculation showing the superposition of the ABCC6 open conformation protein model with its initial template 3G5U (A) and the I-TASSER template (B) individually.** The target structure is colored yellow and the template is in red.
(TIF)

Figure S6 **The sequence alignment of four templates (structure generated from MODELLER, Genesilico, Pcons, I-TASSER) for open conformation model and the ABCC6 structure (query) is shown by position.** The core portion (300 to 600 amino-acid positions) of ABCC6 between two transmembrane bundles was found to be highly conserved among the templates and target sequence. The probable helix beta sheet has been indicated by small boxes; the conserved amino acids are indicated by the red boxes.
(TIF)

Figure S7 **Prediction of transmembrane (TM) topology of the ABCC6 protein model using the PONGO server.** PONGO server (used different programs including MEMSAT, TMHMM2, PRODIV and ENSEMBLE 1.0) revealed the presence of 12 alpha-transmembrane domains (shown in red); 6 of them are located in between 302–600 amino acid residues, the remaining 6 are located in between 945–1225 amino acid residues.
(TIF)

Figure S8 **Structural disordered region of ABCC6 as identified using the MetaDisorder server.** The plot shows the results from a series of predictors including- (a) DISOPRED2 [89], (b) PrDOS [90] (c) IUPred short [91] (d) IUpred long [91] (e) Pdisorder (SoftBerry Product) (f) DISPROT [92] and GlobPlot [93]. The region between amino acids 850–950 was found to be disordered with a Disorder tendency value greater than 0.5.
(TIF)

Figure S9 **Docking results shows that vitamin K3 (red) binds near the transmembrane domain at the outer face of the transmembrane region of open conformation model, thus not binding in any of the predicted substrate binding sites (green).**
(TIF)

Figure S10 **Multiple sequence alignment of human ABCC6 (MRP6), ABCC1 (MRP1) and ABCC2 (MRP2) performed in TCOFFEE (** http://tcoffee.crg.cat/apps/tcoffee/do:regular **).** The conserved amino acid residues involved in substrate binding activities by forming hydrogen bonds are marked by black boxes.
(TIF)

Table S1 **List of compounds reported to be transported in vitro by the human ABCC6 protein.** No: Number.
(DOCX)

Table S2 **The amino acid residues that constitute and support substrate binding site-1 and binding site-2 are shown along with the corresponding transmembrane (TM) domains and chains in the ABCC6 open conformation model.** The interaction of 10 reference compounds with the two substrate binding sites and the hydrogen bonding and hydrophobic interactions involved are indicated by symbols. • = Hydrogen bonding; ??? = Hydrophobic interaction; 1 = BQ-123; 2 = Teniposide; 3 = Etoposide; 4 = Doxorubicin; 5 = NEM-GS; 6 = S-2(2, 4-dinitrophenyl) glutathione; 7 = Daunorubicin; 8 = Leukotriene C4; 9 = vitamin K1; 10 = vitamin K2.
(DOC)
