# Supplementary figures and images for: Molecular Docking Simulations Provide Insights in the Substrate Binding Sites and Possible Substrates of the ABCC6 Transporter
Source: PLoS One. 2014 Jul 25;9(7):e102779. doi: 10.1371/journal.pone.0102779 (PMC4111409; doi:10.1371/journal.pone.0102779)

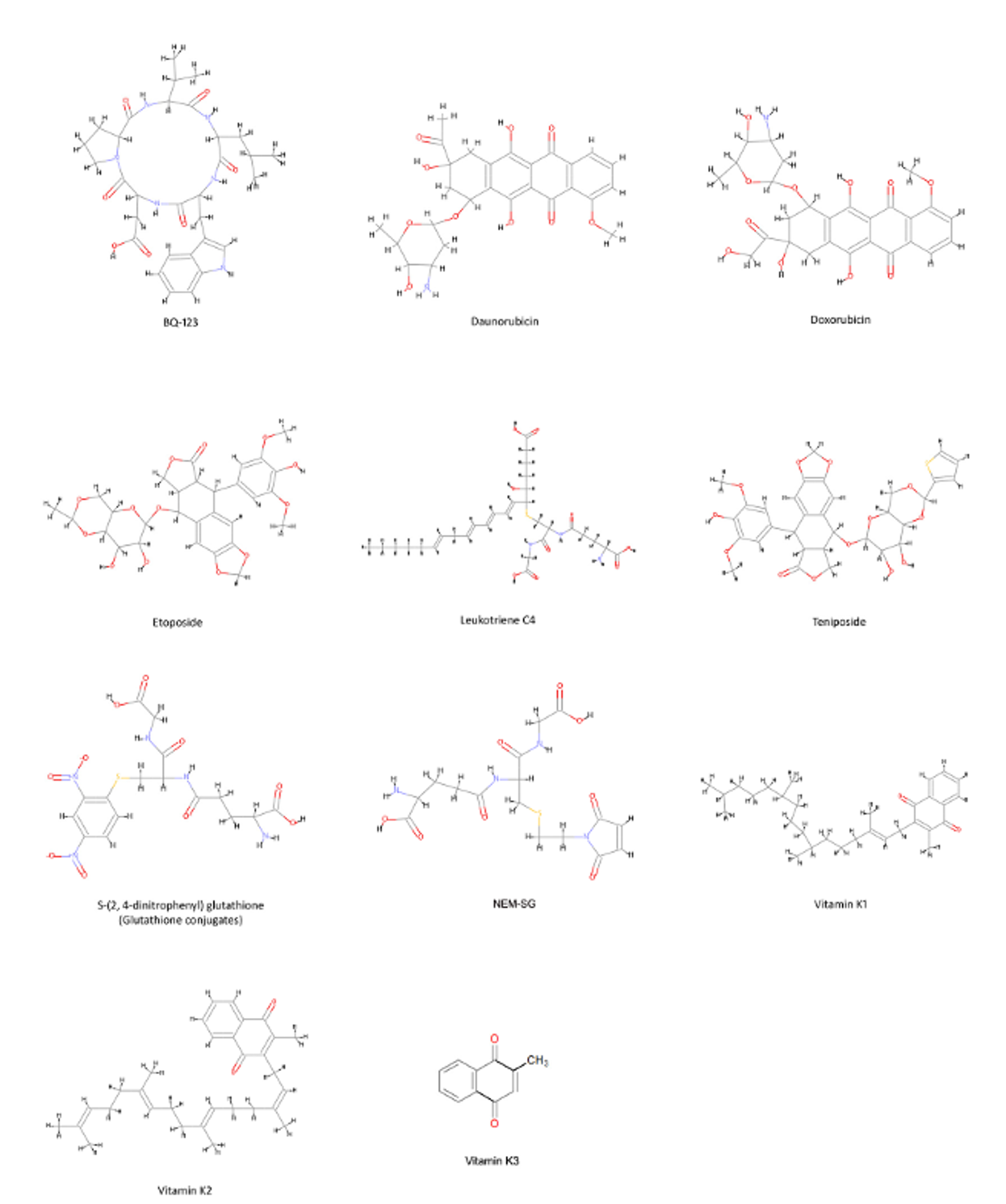

Supplement: Figure S1 — Two dimensional structure of the 8 reported in vitro substrates and 3 vitamin K isoforms. (TIF) [file pone.0102779.s001.tif]

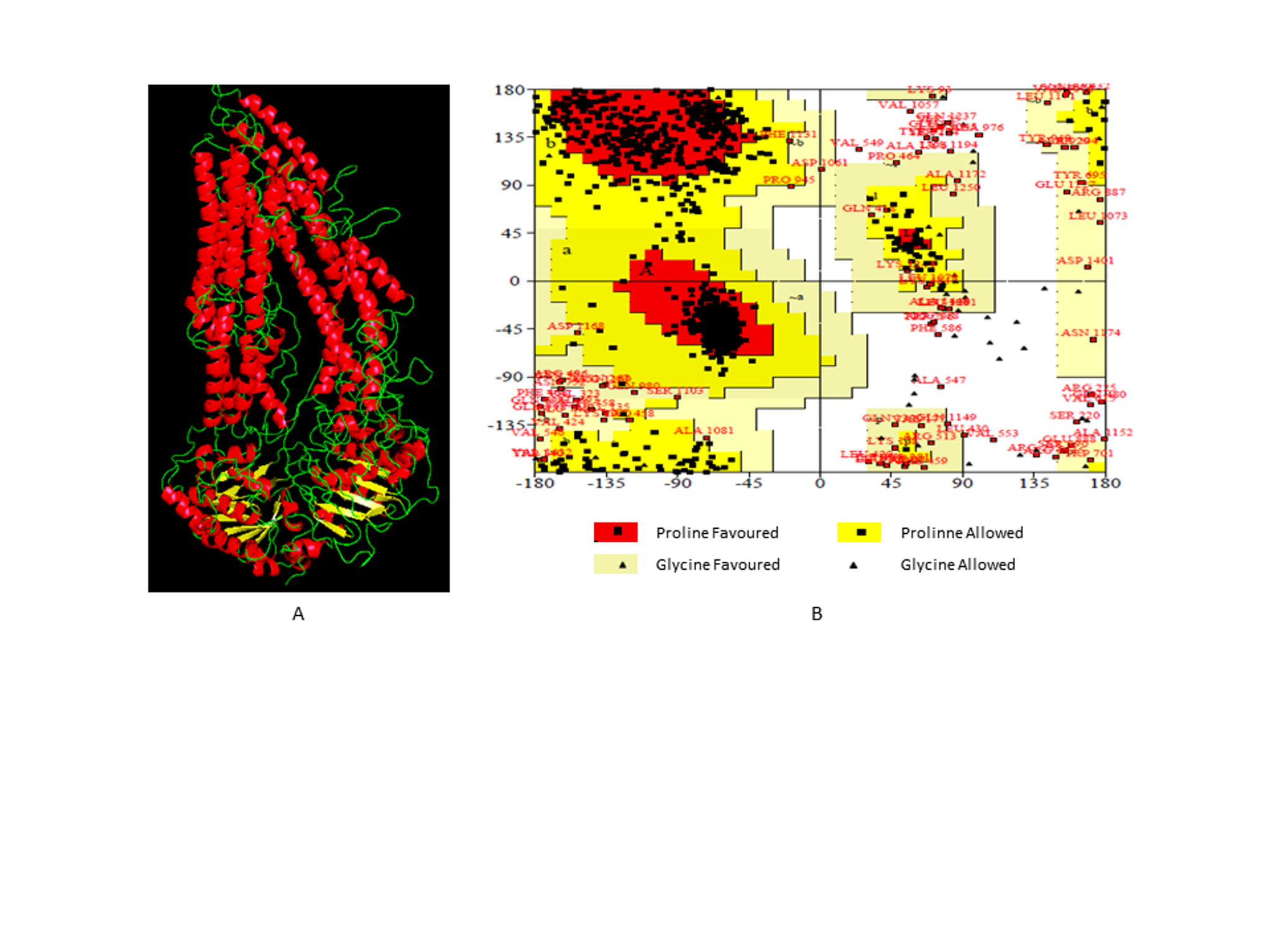

Supplement: Figure S2 — Human ABCC6 protein homology model containing full-length amino acid residues. (A) Ribbon representation of the model in wide open conformation. Helix and sheet regions are shown in red and yellow respectively, whereas the green color represents the loop regions. (B) Ramachandran plot showing distribution of amino acid residues in favored, allowed and disallowed region. (TIF) [file pone.0102779.s002.tif]

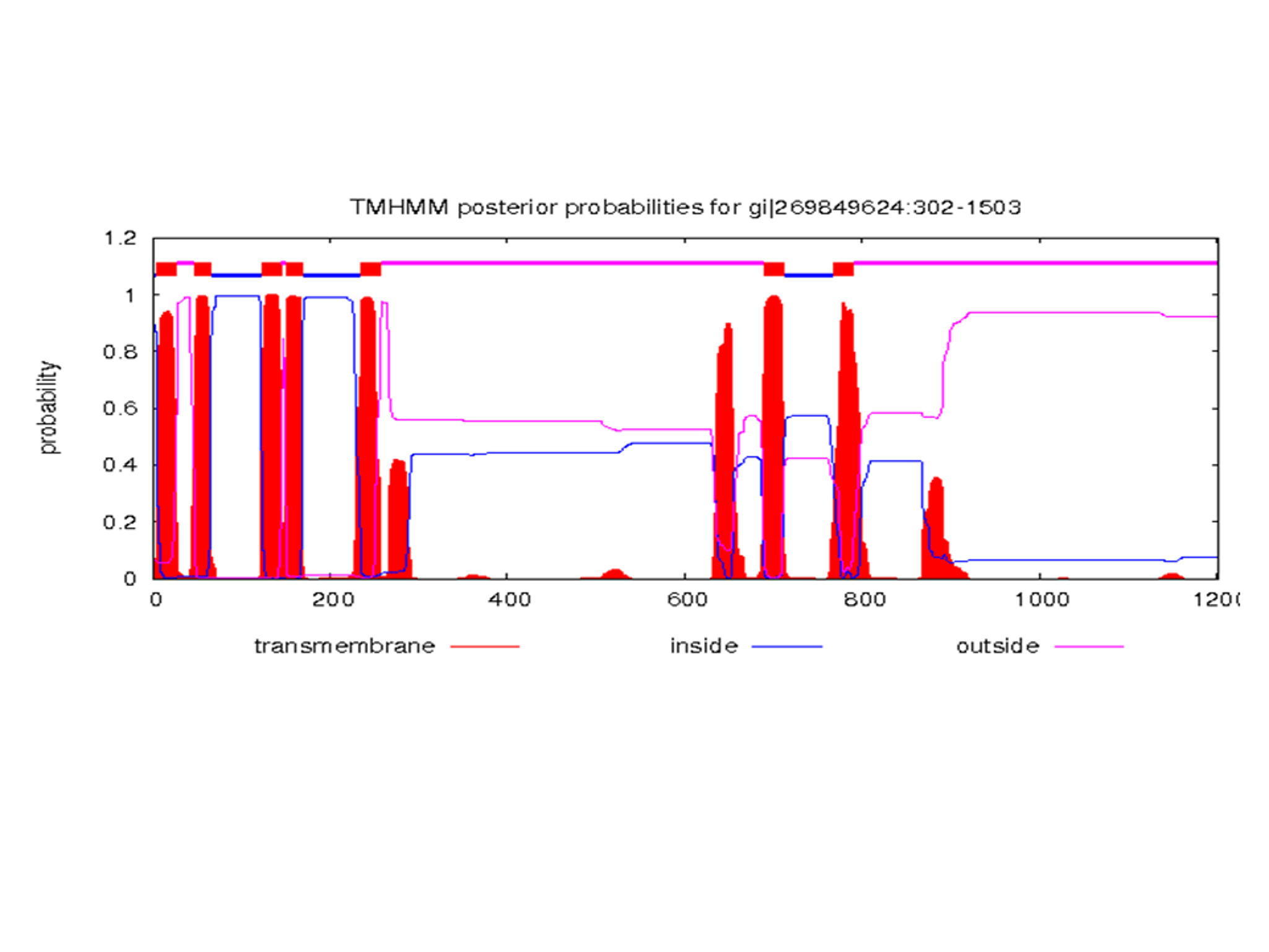

Supplement: Figure S3 — TMHMM plot shows the probability of the presence of TM region in the sequence. It shows the amino acid-wise probabilities for inside, outside and transmembrane region of the ABCC6 sequence. Probability ranges from 0 to 1. At the top of the plot, between 1 to 1.2, the best predictions have been shown as summary. Figure shows 6 TM regions at the first 300 aminoacids, and 4 TM regions from amino acid position 600 to 950. (TIF) [file pone.0102779.s003.tif]

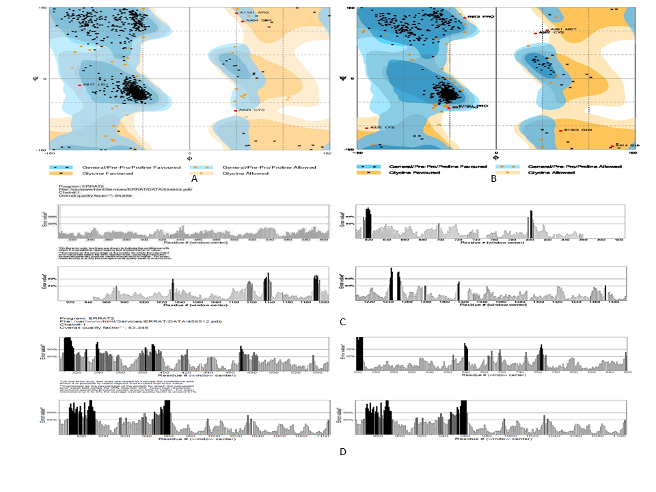

Supplement: Figure S4 — Stereo-chemical validation of the human ABCC6 protein models. Ramachandran Plot generated by the RAMPAGE server shows that- (A) in the open conformation model 96.7% of residues are plotted in the favored region (dark cyan color), 3.0% of residues in the allowed region (cyan color) and 0.4% of residues in the outliner region (red dot), (B) in the closed conformation model 92% of the amino acids residues are plotted in the favored region, 7.1% residues are in the allowed region and 0.3% residues are in the outliner region. (C, D) ERRAT plot for the ABCC6 model as predicted by the ERRAT server. ERRAT showed that the model has an overall quality factor of 94.858% (C) and 83.345% (D) in the open and closed conformation respectively. (TIF) [file pone.0102779.s004.tif]

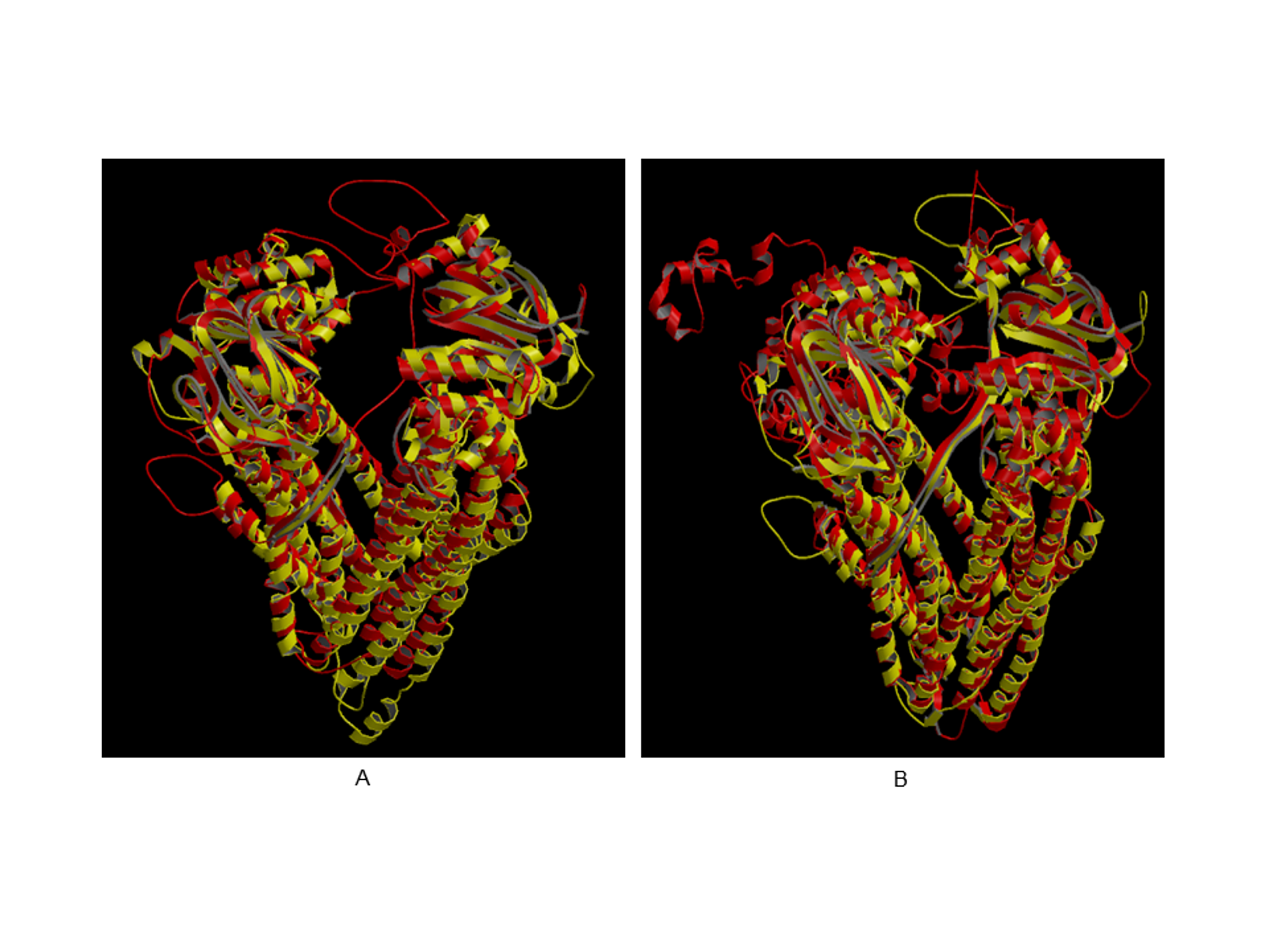

Supplement: Figure S5 — The SuperPose calculation showing the superposition of the ABCC6 open conformation protein model with its initial template 3G5U (A) and the I-TASSER template (B) individually. The target structure is colored yellow and the template is in red. (TIF) [file pone.0102779.s005.tif]

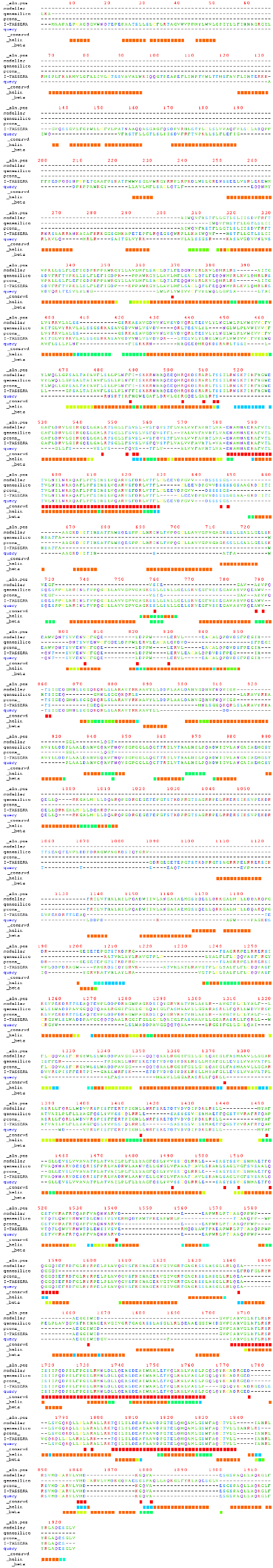

Supplement: Figure S6 — The sequence alignment of four templates (structure generated from MODELLER, Genesilico, Pcons, I-TASSER) for open conformation model and the ABCC6 structure (query) is shown by position. The core portion (300 to 600 amino-acid positions) of ABCC6 between two transmembrane bundles was found to be highly conserved among the templates and target sequence. The probable helix beta sheet has been indicated by small boxes; the conserved amino acids are indicated by the red boxes. (TIF) [file pone.0102779.s006.tif]

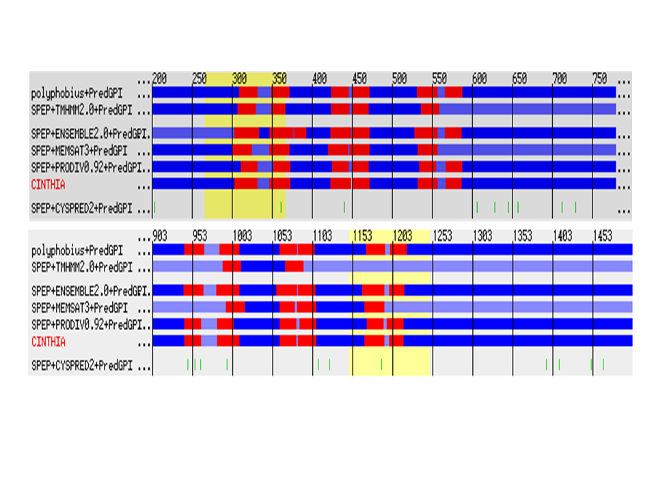

Supplement: Figure S7 — Prediction of transmembrane (TM) topology of the ABCC6 protein model using the PONGO server. PONGO server (used different programs including MEMSAT, TMHMM2, PRODIV and ENSEMBLE 1.0) revealed the presence of 12 alpha-transmembrane domains (shown in red); 6 of them are located in between 302–600 amino acid residues, the remaining 6 are located in between 945–1225 amino acid residues. (TIF) [file pone.0102779.s007.tif]

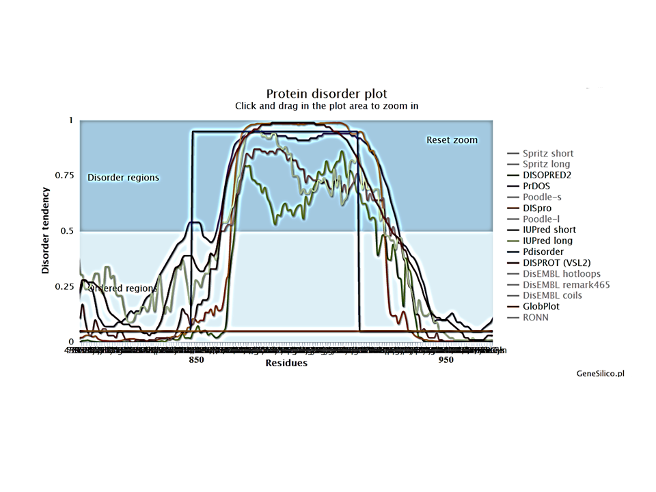

Supplement: Figure S8 — Structural disordered region of ABCC6 as identified using the MetaDisorder server. The plot shows the results from a series of predictors including- (a) DISOPRED2 [89], (b) PrDOS [90] (c) IUPred short [91] (d) IUpred long [91] (e) Pdisorder (SoftBerry Product) (f) DISPROT [92] and GlobPlot [93]. The region between amino acids 850–950 was found to be disordered with a Disorder tendency value greater than 0.5. (TIF) [file pone.0102779.s008.tif]

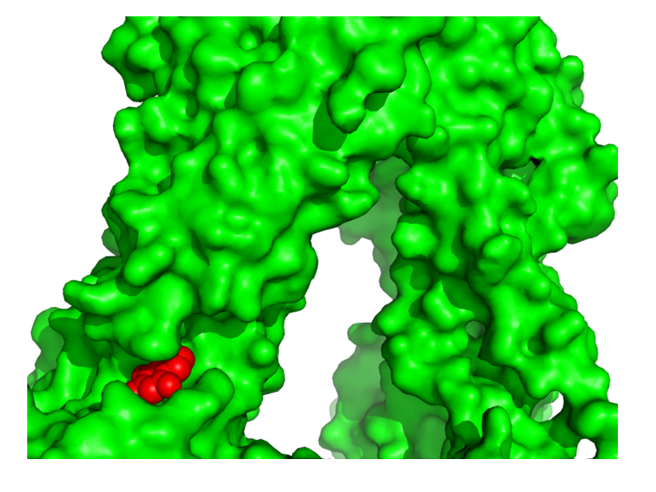

Supplement: Figure S9 — Docking results shows that vitamin K3 (red) binds near the transmembrane domain at the outer face of the transmembrane region of open conformation model, thus not binding in any of the predicted substrate binding sites (green). (TIF) [file pone.0102779.s009.tif]

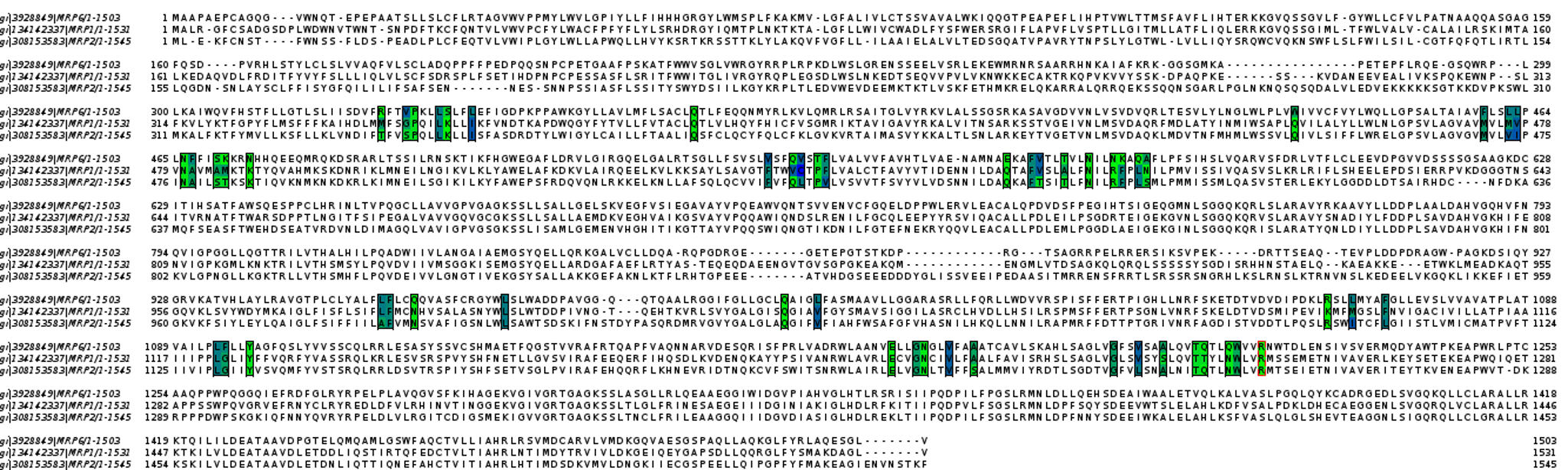

Supplement: Figure S10 — Multiple sequence alignment of human ABCC6 (MRP6), ABCC1 (MRP1) and ABCC2 (MRP2) performed in TCOFFEE ( http://tcoffee.crg.cat/apps/tcoffee/do:regular ). The conserved amino acid residues involved in substrate binding activities by forming hydrogen bonds are marked by black boxes. (TIF) [file pone.0102779.s010.tif]
