# Supplementary material for: Molecular Docking Simulations Provide Insights in the Substrate Binding Sites and Possible Substrates of the ABCC6 Transporter
Source: PLoS One. 2014 Jul 25;9(7):e102779. doi: 10.1371/journal.pone.0102779 (PMC4111409; doi:10.1371/journal.pone.0102779)
Supplement: Table S1 — List of compounds reported to be transported in vitro by the human ABCC6 protein. No: Number. (DOCX) [file pone.0102779.s011.docx]

Table S1. List of compounds reported to be transported in vitro by the human ABCC6 protein. No: Number.

| S. No | Substrates of ABCC6 | References |
| --- | --- | --- |
| 1 | Daunorubicin | [7] |
| 2 | Doxorubicin | [7] |
| 3 | Etoposide | [7] |
| 4 | NEM-GS | [8] |
| 5 | S-2(2, 4-dinitrophenyl) glutathione | [7, 8] |
| 6 | Leukotriene C4 | [7, 8] |
| 7 | Teniposide | [7] |
| 8 | BQ-123 | [7] |
| 9 | Vitamin K1 | [30] |
| 10 | Vitamin K2 | [30] |
