# Supplementary material for: Molecular Docking Simulations Provide Insights in the Substrate Binding Sites and Possible Substrates of the ABCC6 Transporter
Source: PLoS One. 2014 Jul 25;9(7):e102779. doi: 10.1371/journal.pone.0102779 (PMC4111409; doi:10.1371/journal.pone.0102779)
Supplement: Table S2 — The amino acid residues that constitute and support substrate binding site-1 and binding site-2 are shown along with the corresponding transmembrane (TM) domains and chains in the ABCC6 open conformation model. The interaction of 10 reference compounds with the two substrate binding sites and the hydrogen bonding and hydrophobic interactions involved are indicated by symbols. • = Hydrogen bonding; ??? = Hydrophobic interaction; 1 = BQ-123; 2 = Teniposide; 3 = Etoposide; 4 = Doxorubicin; 5 = NEM-GS; 6 = S-2(2, 4-dinitrophenyl) glutathione; 7 = Daunorubicin; 8 = Leukotriene C4; 9 = vitamin K1; 10 = vitamin K2. (DOC) [file pone.0102779.s012.doc]

Table S2. The amino acid residues that constitute and support substrate binding site-1 and binding site-2 are shown in open conformation ABCC6 model, along with the corresponding transmembrane (TM) domains and chains of ABCC6. The interaction of 10 reference compounds with two substrate binding sites and the hydrogen bonding and hydrophobic interactions involved are indicated by symbols.

| **Binding Site-I** | | | | | | | | | | | | | | | | | | | | | | | | | | | | | | | | | | | | |  | |  |  |
| --- | --- | --- | --- | --- | --- | --- | --- | --- | --- | --- | --- | --- | --- | --- | --- | --- | --- | --- | --- | --- | --- | --- | --- | --- | --- | --- | --- | --- | --- | --- | --- | --- | --- | --- | --- | --- | --- | --- | --- | --- |
| **Chain A** | **Substrates involved in hydrogen bonding** | | | | | | | | | | | | **TM** | | | **Chain B** | **Substrates involved in hydrogen bonding** | | | | | | | | | | | | | | | | | | | | **TM** | |  |  |
|  | **1** | | **2** | **3** | 4 | **5** | **6** | **7** | **8** | **9** | **10** | |  | | |  | **1** | | **2** | | **3** | | **4** | **5** | | **6** | | **7** | | **8** | | **9** | | **10** | | |  | |  |  |
| P329 |  | |  |  |  |  |  |  |  |  |  | | 6 | | | L946 |  | |  | |  | |  |  | |  | |  | |  | |  | |  | | | 12 | |  |  |
| L332 |  | |  |  |  |  |  |  |  |  |  | | 6 | | | L953 |  | |  | |  | |  | ☼ | |  | |  | |  | |  | |  | | | 12 | |  |  |
| S333 |  | |  |  |  |  |  |  |  | ☼ |  | | 6 | | | F954 |  | | ☼ | | ☼ | |  | ☼ | |  | |  | |  | |  | |  | | | 12 | |  |  |
| L336 |  | |  |  |  |  |  |  |  |  |  | | 6 | | | Q957 |  | | ☼ | | ☼ | |  | ☼ | | ☼ | |  | |  | |  | |  | | | 12 | |  |  |
| F459 |  | | ☼ | ☼ |  |  |  |  |  |  |  | | 9 | | | S961 |  | |  | |  | |  |  | |  | |  | |  | |  | |  | | | 12 | |  |  |
| L462 |  | | ☼ | ☼ |  |  |  |  |  |  |  | | 9 | | | R964 |  | |  | |  | |  |  | | ☼ | |  | |  | |  | |  | | | 12 | |  |  |
| L463 |  | | ☼ | ☼ |  |  |  |  | ☼ |  |  | | 9 | | | G965 |  | |  | |  | |  |  | |  | |  | |  | |  | |  | | | 12 | |  |  |
| N466 |  | | ☼ | ☼ |  |  | ☼ |  | ☼ |  |  | | 9 | | | L968 |  | |  | |  | |  |  | |  | |  | |  | | ☼ | |  | | | 12 | |  |  |
| F467 |  | | ☼ |  |  |  |  |  | ☼ |  |  | | 9 | | | Q998 |  | | ☼ | |  | |  | ☼ | |  | |  | |  | |  | |  | | | 13 | |  |  |
| S470 |  | | ☼ |  |  |  |  |  | ● |  |  | | 9 | | | L1002 |  | |  | |  | |  | ☼ | |  | |  | |  | |  | |  | | | 13 | |  |  |
| K471 |  | |  |  |  |  |  |  | ☼ |  |  | | 9 | | | S1005 |  | |  | |  | |  |  | |  | |  | |  | |  | |  | | | 13 | |  |  |
| N474 |  | |  |  |  |  |  |  | ● |  |  | | 9 | | | M1006 |  | |  | |  | |  |  | |  | |  | |  | |  | |  | | | 13 | |  |  |
| H475 |  | |  |  |  |  |  |  |  |  |  | | 9 | | | V1009 |  | |  | |  | |  |  | |  | |  | |  | |  | |  | | | 13 | |  |  |
| Q477 |  | |  |  |  |  |  |  |  |  |  | | 9 | | | L1010 |  | |  | |  | |  |  | |  | |  | |  | |  | |  | | | 13 | |  |  |
| L530 |  | |  |  |  |  |  |  |  |  |  | | 10 | | | D1056 |  | |  | |  | |  |  | |  | |  | |  | |  | |  | | | 14 | |  |  |
| S533 |  | |  |  |  |  |  |  |  |  |  | | 10 | | | V1057 |  | |  | |  | |  |  | |  | |  | |  | |  | |  | | | 14 | |  | |
| V534 |  | |  |  |  |  |  |  |  |  |  | | 10 | | | P1060 |  | |  | |  | |  |  | |  | |  | |  | |  | |  | | | 14 | |  | |
| L536 |  | |  |  |  |  |  |  |  |  |  | | 10 | | | D1061 |  | |  | |  | |  |  | |  | |  | |  | |  | |  | | | 14 | |  | |
| V537 |  | | ☼ | ☼ |  | ☼ |  |  |  |  |  | | 10 | | | R1064 |  | |  | |  | |  | ☼ | |  | |  | |  | |  | |  | | | 14 | |  | |
| Q540 |  | | ● | ☼ |  | ☼ | ☼ |  | ☼ |  |  | | 10 | | | L1067 |  | |  | |  | |  | ☼ | |  | |  | |  | |  | |  | | | 14 | |  | |
| V541 |  | | ● | ● |  | ☼ | ☼ |  |  |  |  | | 10 | | | F1071 |  | |  | |  | |  | ☼ | |  | |  | |  | |  | |  | | | 14 | |  | |
| T543 |  | | ☼ | ☼ |  |  | ☼ |  |  |  |  | | 10 | | | G1203 | ☼ | |  | |  | |  |  | |  | |  | |  | |  | |  | | | 17 | |  | |
| F544 |  | | ☼ | ☼ |  | ☼ | ☼ |  |  |  |  | | 10 | | | V1206 |  | |  | |  | |  |  | |  | |  | |  | | ☼ | |  | | | 17 | |  | |
| E565 |  | |  |  |  |  |  |  |  |  |  | | L | | | S1207 |  | |  | |  | |  |  | |  | |  | |  | |  | |  | | | 17 | |  | |
| F568 |  | |  |  |  |  |  |  |  | ☼ |  | | L | | | L1210 |  | |  | |  | |  |  | | ☼ | |  | |  | |  | |  | | | 17 | |  | |
| V569 |  | |  |  |  |  |  |  |  | ☼ |  | | L | | | Q1214 |  | | ☼ | | ☼ | |  | ☼ | | ☼ | |  | |  | |  | |  | | | 17 | |  | |
| T572 |  | |  |  |  |  |  |  |  | ☼ |  | | 11 | | | Q1217 | ● | |  | | ☼ | | ☼ | ☼ | | ● | | ☼ | | ● | |  | |  | | | 17 | |  | |
| N575 |  | | ☼ | ☼ |  | ● | ● |  |  |  |  | | 11 | | | W1218 |  | | ☼ | | ☼ | |  | ☼ | | ● | |  | |  | |  | |  | | | 17 | |  | |
| N578 |  | | ☼ | ● |  |  | ● |  | ☼ |  |  | | 11 | | | R1221 |  | | ☼ | | ● | |  |  | | ☼ | |  | | ☼ | |  | |  | | | 17 | |  | |
| K579 | ● | | ☼ | ☼ |  | ● | ● |  | ● |  |  | | 11 | | | W1223 |  | |  | |  | |  |  | |  | |  | |  | |  | |  | | | 17 | |  | |
| Q581 |  | | ☼ |  |  |  |  |  | ☼ |  |  | | 11 | | |  |  | | | | | | | | | | | | | | | | | | | |  | |  | |
| A582 |  | | ☼ | ☼ |  |  |  |  | ☼ |  |  | | 11 | | |  |  | | | | | | | | | | | | | | | | | | | |  | |  | |
| L584 |  | |  |  |  |  |  |  |  |  |  | | 11 | | |  |  | | | | | | | | | | | | | | | | | | | |  | |  | |
| P585 |  | |  |  |  |  |  |  |  |  |  | | 11 | | |  |  | | | | | | | | | | | | | | | | | | | |  | |  | |
| H589 |  | |  |  |  |  |  |  |  |  |  | | 11 | | |  |  | | | | | | | | | | | | | | | | | | | |  | |  | |
| Binding site-II | | | | | | | | | | | | | | | | | | | | | | | | | | | | | | | | | | | | | | |  | |
| **Chain A** | | **Substrates involved in hydrogen bonding** | | | | | | | | | | | | **TM** | **Chain B** | | | **Substrates involved in hydrogen bonding** | | | | | | | | | | | | | | | | | | | | **TM** |  | |
|  | | **1** | **2** | **3** | **4** | **5** | **6** | **7** | **8** | **9** | | **10** | |  |  | | | **1** | | **2** | | **3** | | | **4** | | **5** | | **6** | | **7** | | **8** | | **9** | **10** | |  |  | |
| R325 | |  |  |  | ☼ |  |  | ☼ |  |  | | ☼ | | 6 | V1079 | | |  | |  | |  | | |  | |  | |  | |  | |  | |  |  | | 14 |  | |
| V328 | |  |  |  |  |  |  |  |  |  | | ☼ | | 6 | V1080 | | |  | |  | |  | | |  | |  | |  | |  | |  | |  |  | | 14 |  | |
| P329 | |  |  |  |  |  |  |  |  | ☼ | | ☼ | | 6 | A1083 | | |  | |  | |  | | |  | |  | |  | |  | |  | |  |  | | L |  | |
| L332 | |  |  |  |  |  |  |  |  | ☼ | | ☼ | | 6 | T1084 | | |  | |  | |  | | |  | |  | |  | |  | |  | |  |  | | L |  | |
| F335 | |  |  |  |  |  |  |  |  |  | |  | | 6 | A1087 | | |  | |  | |  | | |  | |  | |  | |  | |  | |  |  | | 15 |  | |
| L336 | |  |  |  |  |  |  |  |  | ☼ | |  | | 6 | T1088 | | |  | |  | |  | | |  | |  | |  | |  | |  | |  |  | | 15 |  | |
| I339 | |  |  |  |  |  |  |  |  |  | |  | | 6 | L1092 | | |  | |  | |  | | |  | |  | |  | |  | |  | |  |  | | 15 |  | |
| P342 | |  |  |  |  |  |  |  |  |  | |  | | L | L1094 | | |  | |  | |  | | |  | |  | |  | |  | |  | | ☼ |  | | 15 |  | |
| K343 | |  |  |  |  |  |  |  |  |  | |  | | L | F1095 | | |  | |  | |  | | | ☼ | |  | |  | | ☼ | |  | | ☼ | ☼ | | 15 |  | |
| P345 | |  |  |  |  |  |  |  |  |  | |  | | 7 | Y1098 | | | ☼ | |  | |  | | | ☼ | |  | |  | | ☼ | |  | | ☼ | ☼ | | 15 |  | |
| K348 | |  |  |  |  |  |  |  |  |  | |  | | 7 | E1176 | | | ☼ | |  | |  | | | ● | |  | |  | | ● | |  | |  | ☼ | | 16 |  | |
| G349 | |  |  |  |  |  |  |  |  |  | |  | | 7 | G1179 | | |  | |  | |  | | | ☼ | |  | |  | | ☼ | |  | | ☼ | ☼ | | 16 |  | |
| L352 | |  |  |  |  |  |  |  |  |  | |  | | 7 | N1180 | | |  | |  | |  | | | ● | |  | |  | | ☼ | |  | |  | ☼ | | 16 |  | |
| Q363 | |  |  |  | ☼ |  |  | ☼ |  |  | | ☼ | | 7 | L1182 | | |  | |  | |  | | |  | |  | |  | |  | |  | |  |  | | 16 |  | |
| W436 | | ☼ |  |  |  |  |  |  |  |  | |  | | 8 | V1183 | | |  | |  | |  | | | ☼ | |  | |  | | ☼ | |  | | ☼ | ☼ | | 16 |  | |
|  | |  | | | | | | | | | | | |  | A1186 | | |  | |  | |  | | |  | |  | |  | |  | |  | | ☼ | ☼ | | 16 |  | |
|  | |  | | | | | | | | | | | |  | K1194 | | |  | |  | |  | | |  | |  | |  | |  | |  | |  |  | | L |  | |
|  | |  | | | | | | | | | | | |  | S1198 | | |  | |  | |  | | |  | |  | |  | |  | |  | |  |  | | L |  | |
|  | |  | | | | | | | | | | | |  | V1206 | | |  | |  | |  | | |  | |  | |  | |  | |  | |  |  | | 17 |  | |
|  | |  | | | | | | | | | | | |  | A1208 | | |  | |  | |  | | |  | |  | |  | |  | |  | |  |  | | 17 |  | |
|  | |  | | | | | | | | | | | |  | A1209 | | |  | |  | |  | | | ☼ | |  | |  | | ☼ | |  | | ☼ | ☼ | | 17 |  | |
|  | |  | | | | | | | | | | | |  | T1213 | | |  | |  | |  | | | ● | |  | |  | | ● | |  | |  | ☼ | | 17 |  | |

● = Hydrogen bonding.

☼ = Hydrophobic interaction

1 = BQ-123

2 = Teniposide

3 = Etoposide

4 = Doxorubicin

5 = NEM-GS

6 = S-2(2, 4-dinitrophenyl) glutathione

7 = Daunorubicin

8 = Leukotriene C4

9 = Vitamin K1

10 = Vitamin K2
